# High-throughput, fluorescent-aptamer-based measurements of steady-state transcription rates for the *Mycobacterium tuberculosis* RNA polymerase

**DOI:** 10.1093/nar/gkad761

**Published:** 2023-09-22

**Authors:** Drake Jensen, Ana Ruiz Manzano, Maxwell Rector, Eric J Tomko, M Thomas Record, Eric A Galburt

**Affiliations:** Department of Biochemistry and Molecular Biophysics, Washington University School of Medicine, Saint Louis, MO 63108, USA; Department of Biochemistry and Molecular Biophysics, Washington University School of Medicine, Saint Louis, MO 63108, USA; Department of Biochemistry, University of Wisconsin, Madison, WI 53706, USA; Department of Biochemistry and Molecular Biophysics, Washington University School of Medicine, Saint Louis, MO 63108, USA; Department of Biochemistry, University of Wisconsin, Madison, WI 53706, USA; Department of Biochemistry and Molecular Biophysics, Washington University School of Medicine, Saint Louis, MO 63108, USA

## Abstract

The first step in gene expression is the transcription of DNA sequences into RNA. Regulation at the level of transcription leads to changes in steady-state concentrations of RNA transcripts, affecting the flux of downstream functions and ultimately cellular phenotypes. Changes in transcript levels are routinely followed in cellular contexts via genome-wide sequencing techniques. However, *in vitro* mechanistic studies of transcription have lagged with respect to throughput. Here, we describe the use of a real-time, fluorescent-aptamer-based method to quantitate steady-state transcription rates of the *Mycobacterium tuberculosis* RNA polymerase. We present clear controls to show that the assay specifically reports on promoter-dependent, full-length RNA transcription rates that are in good agreement with the kinetics determined by gel-resolved, α-^32^P NTP incorporation experiments. We illustrate how the time-dependent changes in fluorescence can be used to measure regulatory effects of nucleotide concentrations and identity, RNAP and DNA concentrations, transcription factors, and antibiotics. Our data showcase the ability to easily perform hundreds of parallel steady-state measurements across varying conditions with high precision and reproducibility to facilitate the study of the molecular mechanisms of bacterial transcription.

## Graphical Abstract

**Graphical Abstract:**
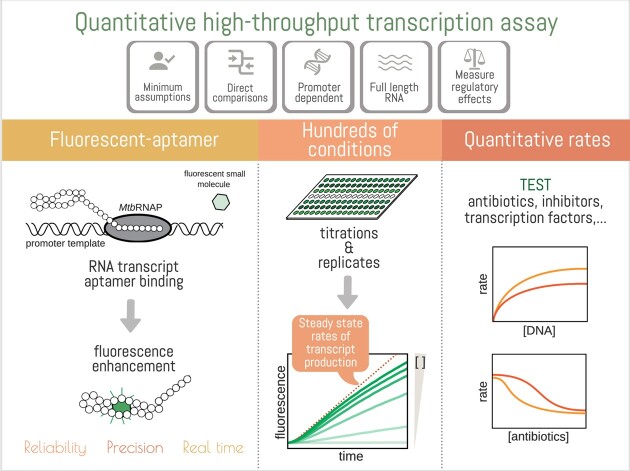

## INTRODUCTION

Cellular RNA abundance is dictated by the relative steady-state rates of RNA production and degradation. In particular, the rate of RNA production is ubiquitously the target of gene regulatory mechanisms and often represents a good proxy for protein synthesis flux (1). More specifically, the rate at which full-length RNA transcripts are generated is typically controlled by the rate of transcription initiation. This is because the overall initiation rate is often slower than subsequent elongation and termination steps, and because multiple RNA polymerases (RNAPs) may be elongating at the same locus at the same time. Bacterial transcription initiation progresses through several intermediates, where the rates and equilibrium constants that describe the initial binding of the RNAP to the promoter and the subsequent isomerization steps that culminate in opening of promoter DNA can vary greatly depending on the sequence of the promoter (2,3). For example, *Escherichia coli* promoters can differ in rates of promoter opening by factors of 10^3^–10^4^, resulting in initiation events ranging from once per second to once per generation (4,5). Following promoter opening, binding of the first two initiating nucleoside triphosphates (NTP) forms an initially transcribing complex that begins producing a nascent RNA transcript. After formation of the initiating dinucleotide, each step of RNA synthesis requires translocation, stressing RNAP–promoter contacts (6,7). If this stress disrupts these contacts, the RNAP escapes from the promoter and begins processive elongation. Otherwise, complexes stall near the promoter or perform cycles of abortive transcription (5,8,9). After decades of careful biophysical dissection, driven mainly by pre-steady-state kinetic and structural biology approaches, bacterial initiation pathways are well-characterized on a handful of different promoters for model bacteria (reviewed in (2,3,10–12)). Many kinetic/structural intermediates have been identified, including off-pathway states that, in some cases, do not lead to full-length RNA production (8,13–17). The resulting models from all these studies are complex and can vary depending on the system studied (18–20), bringing into question model generalizability across bacteria and different promoter sequences.

Ideally, rate constants for RNAP binding, DNA opening, initial transcription and promoter escape obtained from pre-steady-state kinetic measurements could be used to calculate an overall initiation rate or the average time between initiation events. A demonstration of this can be seen in approaches where both basal and regulated RNA flux is calculated based on simple models of transcription initiation (3,21). However, while these models may address theoretical links between initiation kinetics and steady-state rates of RNA production, they cannot account for the large number of variables that determine the rate of transcription. This overall initiation rate is most simply described using Michaelis-Menten enzyme kinetics, where the RNA polymerase is the enzyme, promoter DNA is the substrate, and the full-length RNA transcript is the product (22). This model assumes a constant concentration of RNAP–promoter (enzyme–substrate) complexes generated by a balance between promoter binding and escape rates and predicts a constant reaction velocity or rate of transcript production. Within this formalism, regulated promoters are activated or repressed via changes in the Michaelis-Menten parameters *K_m_* (the concentration at which the half maximal rate is observed) and/or *V_max_* (the maximal rate observed under saturating conditions) without changes in the free RNAP concentration. Alternatively, constitutively active promoters are affected by cell growth-rate-dependent changes in the free RNAP concentration, independent of changes in *K_m_* and/or *V_max_* (23–25). Classic examples of environmental adaptation in bacteria affecting transcription initiation processes include the up-regulation of beta-galactosidase in response to the presence of lactose and the absence of glucose (26), and the genome-wide response to starvation known as the stringent response (27,28).

*In vitro* measurements of steady-state transcription rates can be used to test and develop more complex models but have been limited by current methodologies. Historically, measurements of *in vitro* basal and regulated transcription initiation kinetics have been made possible by monitoring RNA production via the incorporation of radio-labelled NTPs, resolved via polyacrylamide gel electrophoresis (29). The approach was used in the initial discovery of RNAP holoenzyme activity more than fifty years ago (30) and is the most common assay for probing transcriptional mechanisms *in vitro*. The strengths of the assay include its high sensitivity and single-nucleotide resolution even with small quantities of RNA. This allows for the separation of RNA products by length and to investigate individual steps in nucleotide addition reactions that underlie the fundamental mechanisms of RNAP. However, steady-state ^32^P-detected transcription assays have several drawbacks. A practical limitation is that the use of radioactivity is expensive, both for the purchase of the reagent itself and for the requirement of specialized protocols for safe shipping and use in the lab. The reaction must also be taken through several steps that may include the incorporation of proteases or chelators to quench the reaction at set timepoints, phenol/chloroform extractions, and/or denaturation and loading the sample on an electrophoretic polyacrylamide gel. All these steps, as well as the quantification of resolved product bands via image analysis, can introduce non-biochemical variability in the measured RNA amounts. In addition, these assays require significant amounts of time and training, where single experiments often take multiple days before complete quantification and where technical practice is needed for generating reproducible data. All these factors combined result in relatively limited throughput. As a result, the time-dependent measurements needed to quantitate steady-state initiation rates are infrequently performed and single time-point measurements are often used to infer mechanisms of gene regulation.

In contrast to the *in vitro* approaches described above, *in vivo* transcription studies have undergone a remarkable transformation where genome-wide transcript levels in cells under varying environmental and genetic backgrounds are routinely queried (31). We were motivated by the desire to increase the throughput of *in vitro* transcriptional studies to facilitate the efficient testing of mechanistic hypotheses, the development of predictive transcription initiation models, and to be able to compare transcript flux from reconstituted systems to the genome-wide information accessible via RNAseq. To this end, we explored the use of a fluorescent-aptamer-based detection system to report on *in vitro* steady-state transcription rates.

**Figure 1.**
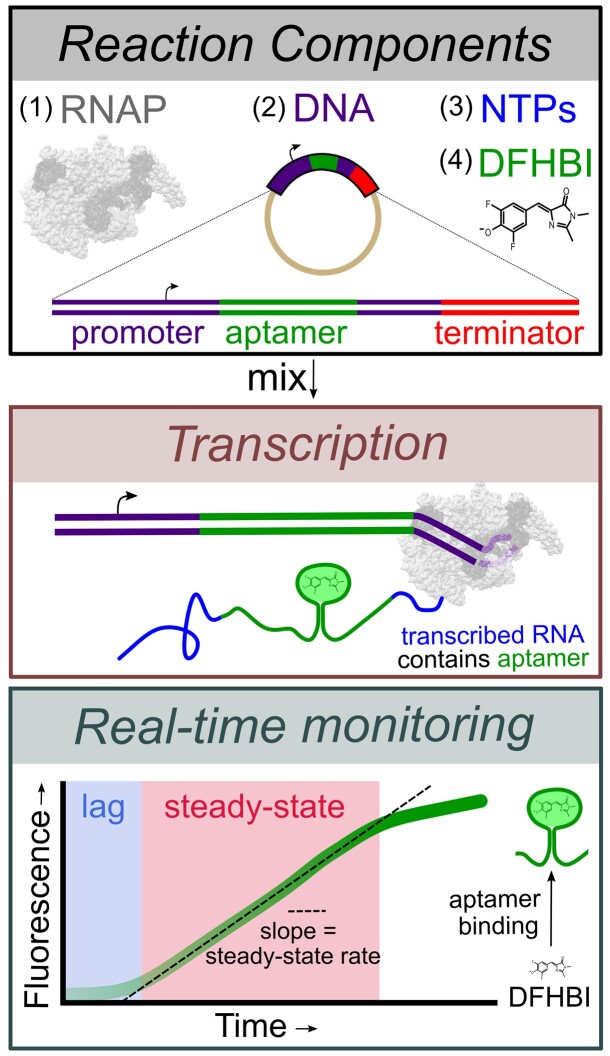
Overview of the fluorescent-aptamer-based assay. This assay requires RNAP, a DNA template containing the sequence for the Spinach-mini RNA aptamer (33), NTPs and the fluorescent dye DFHBI. Upon initiating the reaction, full-length RNAs containing the Spinach-mini aptamer capable of binding DFHBI are synthesized. The fluorescent signal change, monitored in real-time, results from the unbound to aptamer-bound dye transition and is used as a reporter of full-length transcription rates. An initial lag time is observed, followed by a steady-state regime where the slope of the linear phase represents the steady-state rate of full-length RNA synthesis.

Fluorescent light-up aptamers are RNA sequences that bind small molecule fluorophore dyes and generate a large fluorescence enhancement. They have been used in numerous applications (reviewed in (32)) including the detection of nascent RNA transcripts in cells (33–38), in synthetic biology transcription-translation coupled *in vitro* systems for T7 RNAP (39–41) and with cell lysates from diverse bacterial species (42). These approaches rely on the use of a DNA template that encodes for an aptamer sequence so that each transcription event results in the generation of an RNA aptamer which folds and binds the dye. For each transcript produced, there is an accompanying increase in fluorescence (Figure 1). Importantly, as the change in dye fluorescence requires the synthesis of a full-length RNA transcript containing the aptamer, the fluorescent readout is not complicated from short abortive products that may be generated during promoter escape. Using this approach, recent work has illustrated how the rates of *in vitro* reactions can be quantified in a fluorimeter cuvette with *E. coli* RNAP (43) and in a plate-reader format with T7 RNAP (44). Here, we follow up on these studies and provide a description of essential control experiments needed to clearly link the fluorescent signal with the transcription of a promoter-derived product. Additionally, using data acquired with RNAP from *Mycobacterium tuberculosis* (*Mtb*), we describe a workflow for the calibration and quantification of RNA concentration in multi-round initiation kinetics analyzed with a Michaelis-Menten approach. Most significantly, we illustrate the utility of this approach for the investigation of mechanisms of transcriptional regulation by NTP concentration, transcription factors, and antibiotic inhibitors, all derived from high-throughput measurements under steady-state conditions.

## MATERIALS AND METHODS

### Reagents

#### Preparation of DNA constructs

Circular plasmid templates, 2557 base-pair (bp) in length, were ordered from Twist Bioscience (San Francisco, CA), containing the sequences corresponding to the *Mtb* ribosomal RNA promoter (*rrnA*P3), the Spinach-mini aptamer (33) and the *E. coli rrnB*P1 *T*_1_ terminator (45) (illustrated in Figure 1). Plasmids were isolated via the Qiagen Midi Prep Kit (catalogue #: 27106) and were predominantly supercoiled (Supplementary Figure S1). For additional sequences and further descriptions, see Supplementary Table S1. Linear PCR templates 250 bp in length were amplified from 200 nucleotide (nt) PAGE purified DNA Ultramer oligos (Integrated DNA Technologies, Inc., Coralville, IA) following annealing and end-filling. For more details including primer and final construct sequences, as well as further preparation details, see Supplementary Table S2.

#### Preparation of recombinant proteins

The *Mtb* RNAP σ^A^ holoenzyme complex was co-expressed and purified using an N-terminal His-tagged σ^A^ subunit via nickel affinity chromatography, followed by size exclusion chromatography, in accordance with previous methods (18,46). *Mtb* CarD and RbpA, in pET-SUMO plasmid vectors, were expressed and purified, and the His-SUMO tag was subsequently cleaved in accordance with methods previously described (18,46). *E. coli* RNAP σ^70^ holoenzyme was purchased from New England BioLabs, Inc. (Ipswich, MA; catalogue # M0551S). Purified *E. coli* GreB was a generous gift from Irina Artsimovitch (The Ohio State University) (47). Protein concentrations were determined using the following extinction coefficients at 280 nm: *Mtb* RNAP σ^A^ holoenzyme (280,452 M^−1^ cm^−1^), *Mtb* CarD (16,900 M^−1^ cm^−1^), *Mtb* RbpA (13,980 M^−1^ cm^−1^), and *E. coli* GreB (36,900 M^−1^ cm^−1^).

#### Reagents for transcription assays

All transcription reactions were performed with NTPs (Thermo Scientific), 3,5-difluoro-4-hydroxybenzylidene imidazolinone (DFHBI) dye (Sigma Aldrich; catalogue # SML1627), and RiboLock RNase inhibitors (Thermo Scientific; catalogue # EO0381). DFHBI concentration was determined using an extinction coefficient at 420 nm of 31,611 M^−1^ cm^−1^. Rifampicin (Sigma-Aldrich; catalogue #R3501) and Fidaxomicin (Selleckchem; catalogue # S4227) solids were dissolved in DMSO and molar concentrations were determined by weight (Fidaxomicin) or by absorbance using an extinction coefficient at 470 nm of 15,300 M^−1^ cm^−1^ (48) (Rifampicin).

### Plate-reader fluorescence measurements

To measure multi-round kinetics under steady-state conditions in real-time, we monitored the change in DFHBI fluorescence upon binding to a transcribed, full-length RNA sequence containing the Spinach-mini aptamer. Data was collected using a Synergy2 multi-detection microplate reader (BioTek Instruments, Inc., Winooski, VT) with the corresponding Gen5 analysis software. DFHBI fluorescence was measured with a tungsten light source equipped with a 505 nm long-pass dichroic mirror, excited with a 460 ± 40 nm bandpass filter, and the resulting emission signal was monitored with a 528 ± 20 nm bandpass filter. Data was acquired at a read height of 7.00 mm, typically in 20–30 s intervals, with varying total acquisitions times, not exceeding 75 min.

Transcription reaction master mixes containing 90% of the final volume included the RNAP holoenzyme, NTPs, DFHBI, and RNase inhibitors. Based on the volumes added for each corresponding buffer addition and concentrated stock component, the final solution conditions were 20 mM Tris (pH 8.0 at 25°C), 40 mM NaCl, 75 mM K-glutamate, 10 mM MgCl_2_, 5 μM ZnCl_2_, 20 μM EDTA, 5% (v/v) glycerol (defined as transcription buffer) with 1 mM DTT and 0.1 mg/mL BSA. From these master mixes, a volume of 9 μl for each technical replicate was transferred to an individual well in a 384 well, low volume, round-bottom, non-binding polystyrene assay plate (Corning; catalogue # 4514). For the negative controls, which represent the entirety of the reaction components except for DNA, 1 μl of transcription buffer was added to account for the remaining 10% of the final reaction volume (10 μl). Wells were covered with an optical adhesive to prevent sample evaporation (Applied Biosystems; catalogue # 4360954). The plate was then incubated for 10 min at 25°C, followed by a 30 s shake agitation in the microplate reader. An initial reading of the negative controls was used to scale the automatic gain adjustment to the background signal with an arbitrary value of 1000 RFUs and was applied to all the subsequent reads. The adhesive cover was removed, and the transcription reaction was initiated with 1 μl DNA unless otherwise indicated. Multichannel pipettors were used to reduce the initiation time difference across wells. Once the DNA was added, the plate was agitated for 15 sec before starting the kinetic measurements. Unless otherwise stated, the reaction master mixes always contained 20 μM DFHBI and 0.4 U/μl RNase inhibitors. For additional details regarding reaction specifics for individual titrations, including titrations measured under single-round conditions, see Supplementary Materials and Methods.

### Radio-labelled NTP incorporation transcription experiments

Transcription experiments were performed with 500 μM each NTP under identical solution conditions and temperature as multi-round fluorescence experiments. *Mtb* σ^A^ RNAP holoenzyme and DFHBI concentrations were varied and always included unless otherwise indicated. 20 nM of α-^32^P UTP was added to label the nascent RNA transcripts via incorporation by RNAP. Reactions were initiated by addition of circular plasmid constructs containing either 5 nM *Mtb rrnA*P3 or a template lacking a promoter (promoterLESS, see Supplementary Table S1). 5 μl aliquots were removed from the reaction mixture at 1, 5, 10, 15, 20 and 30 min and combined with 5 μl of quench solution containing 95% formamide, 0.015 M EDTA, 0.05% (w/v) xylene cyanol, and 0.05% (w/v) bromophenol blue. 5 μl of each quenched timepoint was then loaded onto a 5% denaturing PAGE gel. Gels were run in TBE for 2 to 3 h at 1,500 V and then transferred to a phosporimaging cassette. After exposing for 18 h, phosphorimaging screens were imaged via a Typhoon 9000 phosphorimager. Bands of interest were quantified using the ImageQuant software and converted to reaction RNA concentrations as previously described (5).

### Analyses of fluorescent time courses

#### Extraction of steady-state rates

As the fluorescent signal in the absence of aptamer formation does not significantly change across conditions where DNA or NTPs were left out of the reaction (Supplementary Figure S3), the same negative control could be used to correct all experimental conditions within a titration. This applies only if the time course measurements are made under the same solution conditions and instrumental detection scaling. However, to minimize experimental and/or instrumental variation, each time a new experiment was performed, a minimum of 2 to 3 technical replicates of the negative control (leaving out DNA) were collected and measured concurrently with the experimental data. Prior to data analysis, the experimental traces underwent two subtractions: first, the fluorescent value recorded at the initial timepoint was subtracted from all timepoints, bringing the starting fluorescent value to zero, and second, the fluorescence from the experimental traces was subtracted using the corresponding signal from each time-point of the negative control.

Linear regression of the corrected fluorescent traces was performed with a custom MATLAB fitting program (described further in Results). Using a statistical weighting from multiple technical replicates per condition and a user inputted *R*^2^ value to define the goodness of fit (Supplementary Figure S6), an unbiased determination of the linear regime can be obtained, where the slope of the fitted line reports on the steady-state rate in units of change in fluorescence/time. In the work presented here, *R*^2^-thresholds >0.998 were used.

#### Non-linear regression analyses of concentration dependencies

For analyses of titration data that yielded hyperbolic concentration dependencies in steady-state rates, a Michaelis-Menten equation was applied, fitting the data to Equation 1


(1) \begin{equation*}v = \ \frac{{{V}_{max}\left[ S \right]}}{{{K}_m + \left[ S \right]}}\end{equation*}

where *v* represents the steady-state rate and *S* represents the titrated substrate, either DNA or individual NTPs. Steady-state rates determined in some NTP titrations were not well-described by Equation 1, displaying sigmoidal concentration dependences. These titrations were fit to Equation 2, a modified Michaelis-Menten equation with three parameters: *V_max_*, an apparent *K_m_* (*K_m_*_,_*_app_*), and the exponent *n*.


(2) \begin{equation*}v = \ \frac{{{V}_{max}{{\left[ {NTPs} \right]}}^n}}{{{K}_{m,ap{p}^n} + {{\left[ {NTPs} \right]}}^n}}\end{equation*}

Here, we define *K_m_*_,_*_app_* as the NTP concentration that results in half maximal velocity, which is equivalent to the standard operational definition of *K_m_* when *n* = 1. It should be noted that while this equation is functionally identical to the Hill equation, if a non-unity *n* is the result of the kinetics from a single-site-binding system, then *n* is only effectively demonstrative of the type (positive/negative) and magnitude of cooperativity. In such a case, *n* has no specific physical meaning as it does in more traditional cases such as cooperative binding of multiple ligands (49). The kinetics of transcription inhibition as a function of antibiotic concentration at constant NTP concentrations were also found to be sigmoidal. For determination of the half maximal inhibitory concentration (*IC_50_*) of Rifampicin and Fidaxomicin, the concentration-dependent reduction in steady-state rates was fit to Equation 3.


(3) \begin{equation*}v = \ \frac{{{V}_{max}{{\left[ {Antibiotic} \right]}}^n}}{{IC_{50}^{\ n} + {{\left[ {Antibiotic} \right]}}^n}}\end{equation*}

### Statistical analyses

For all fluorescent data presented unless otherwise indicated, between 2 and 4 independent experiments were collected for each condition tested. Within each independent experiment, standard deviations from 2 to 3 technical replicates were used as a statistical weight during the linear regression analyses. All non-linear regression analyses were performed using the standard deviations of the independent experiments as a statistical weight. Errors in *V_max_*, *K_m,app_*, *n* and *IC_50_* values are those obtained from the fit of the averaged data set. For simplicity, only a single representative independent experiment is shown in the plots. For gel-based quantifications, average values and standard deviations from 2 to 3 independent experiments are reported.

### Data availability

The MATLAB code used for automated linear regression analyses has been made publicly available and can be accessed with the following GitHub link (https://github.com/egalburt/aptamer-flux-fitting).

## RESULTS

### Promoter-dependent, aptamer-based measurements of full-length transcription

As similar assays have been described elsewhere, we briefly describe the specifics of the version we utilized for these studies. DNA templates were constructed such that an 80 nt Spinach-mini aptamer sequence (33) was inserted 30 bp downstream of the +1 transcription start site corresponding to the genomic *Mtb* ribosomal RNA promoter sequence (*rrnA*P3). Specifically, –60 to +31 of *rrnA*P3 was included to account for any upstream-promoter interactions as well as the initially transcribed sequences, both of which can regulate initiation kinetics. Inclusion of the genomic initially transcribed sequence, rather than just positioning the aptamer sequence to start at the +1 position, provides for unaltered promoter escape, which is known to become rate limiting under certain conditions (see (3) for examples of sequence effects on initiation kinetics). The *E. coli* intrinsic *rrnB*P1 *T*_1_ terminator sequence was included in circular plasmid DNA templates to dissociate the ternary polymerase, template, and transcript complex. Sequences for both circular plasmid and linear PCR templates are given in Supplementary Tables S1 and S2.

When transcribed downstream of the *Mtb rrnA*P3 promoter, the Spinach-mini aptamer sequence folds and binds the small molecule fluorophore DFHBI, resulting in a fluorescence enhancement. By mixing RNAP, DNA, all NTPs and DFHBI, the reaction is monitored in real-time where the increase in fluorescence reports on transcript production. All experiments were performed in a 384 well plate-reader format using 10 μl reaction volumes facilitating high-throughput measurements of steady-state initiation kinetics with minimal sample volume requirements. The slope of the fluorescent signal at early times reports on the steady-state rate of transcription initiation (Figure 1). In general, one expects initiation kinetics and not co-transcriptional aptamer folding (39,50,51) or dye binding (52) to be rate-limiting for functional aptamer production. However, even when the initiation rate is not limiting relative to the timescales of elongation or aptamer folding, the steady-state rate will still specifically report on the initiation rate (Supplementary Figure S2). This is because once one RNAP leaves the promoter, another RNAP may bind and begin the process of initiation irrespective of these downstream processes. This condition may be broken by pathological cases where downstream pausing creates a traffic jam that backs up onto the promoter, influencing the time needed for the next polymerase to bind the promoter.

**Figure 2.**
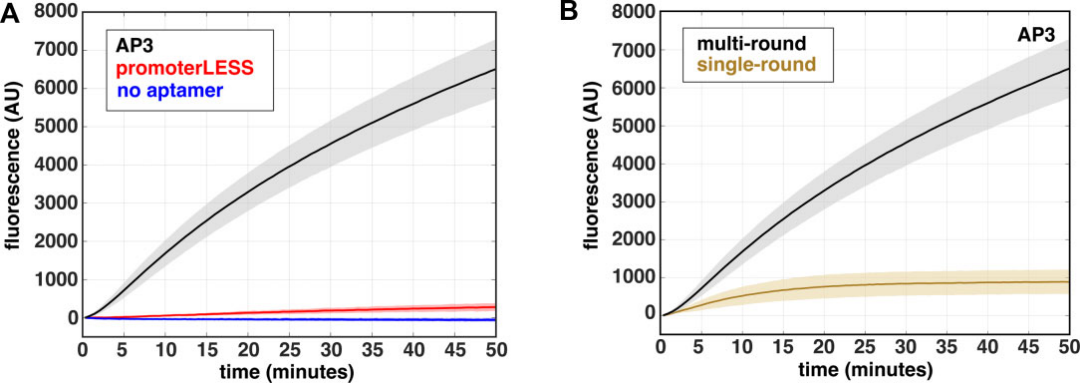
Real-time fluorescent signal is promoter dependent and due to multiple rounds of initiation. All experiments were performed using 100 nM *Mtb* RNAP, 5 nM DNA circular plasmid templates, and 500 μM NTPs. (**A**) Comparison of time courses of RNA synthesis from an *Mtb* plasmid template containing either both the *rrnA*P3 and aptamer sequences (black), the *rrnA*P3 sequence with no aptamer sequence (blue), or the ‘promoterLESS’ template, containing the aptamer, but lacking the *rrnA*P3 sequence (red). (**B**) Real-time traces comparing multi-round (black) and single-round conditions (gold) on the *rrnA*P3 circular plasmid template. Error shading indicates the standard deviation of 3 independent experiments.

Transcription reactions were initiated by adding DNA templates to *Mtb* RNAP preincubated with NTPs, DFHBI dye and RNase inhibitors. Fluorescence was monitored in real-time, where we observed three distinct kinetic phases to the trace using the circular plasmid template containing the *rrnA*P3 sequence: (i) a lag time, (ii) a linear increase and (iii) roll-off from the linear regime that begins to plateau over time (Figure 2A, black curve). We note that when starting from a well-controlled time zero, the lag time theoretically reports on the time required for pioneering RNAPs to complete the initiation process, transcribe the aptamer, and for dye to bind the folded RNA transcript (43) (Supplementary Figure S2). In our assay setup, since initiation of the reaction was done by hand, we do not attempt to quantitate the lag time, but focus on the linear regime. In the absence of either DNA or NTPs, only a slow decay in DFHBI fluorescence was observed as a function of time (Supplementary Figure S3), indicating that the increase in fluorescence when all reaction components are present is due to transcription-derived aptamer formation. For all plots shown, the signal in the absence of transcription is subtracted from the experimental traces so that the y-axis reports specifically on the fluorescence generated by transcription of the aptamer (Materials and Methods). Correcting for this time-dependent fluorescent change of the background signal is especially important when evaluating conditions of minimal transcript generation (Supplementary Figure S3).

To confirm that transcription is driven by the *rrnA*P3 promoter sequence, we performed the assay using a circular plasmid template lacking the promoter (i.e. promoterLESS; for sequence, see Supplementary Table S1) as a negative control. The signal generated in the presence of *rrnA*P3 is more than 20-fold higher than that obtained with the promoterLESS template (Figure 2A, red), confirming that the signal arises from promoter-driven transcription. However, the amount of signal generated by the promoterLESS template is not zero, as can be seen by comparing to a trace using a template that contains the promoter but lacks the aptamer sequence (Figure 2A, blue). This comparison suggests that even in the absence of a promoter sequence, some basal level of aptamer is transcribed (see below for further discussion).

### Fluorescence can be monitored under both multi- and single-round conditions

Our specific goal was to obtain measurements of steady-state rates of transcription. We therefore needed to confirm that the observed signal is due to multiple rounds of transcription. We expect significantly higher signal amplitude under multi-round conditions compared to single-round conditions, where DNA traps are used to prevent RNAP rebinding. The preincubation of the reaction with salmon-sperm DNA, which does not actively dissociate RNAPs from the DNA like other competitors such as heparin (20), resulted in the absence of any signal upon the addition of NTPs (Supplementary Materials and Methods and Supplementary Figure S4A). This result shows that the competitor DNA acts as an effective trap for the *Mtb* RNAP and that its inclusion establishes single-round conditions. Upon initiating the transcription of pre-bound RNAP-DNA complexes with 500 μM NTPs and salmon-sperm DNA competitor, we observed no lag time, and the fluorescence rapidly reached saturation as RNAP re-binding was prevented (Figure 2B). These results are consistent with previous reports of single-round conditions for full-length transcript production (9,14,16). In the absence of competitor under otherwise identical conditions, we observed a much larger increase in the overall fluorescent signal (Figure 2B), confirming that in the absence of DNA trap, the assay is multi-round.

In theory, use of this assay under single-round conditions with pre-formed RNAP-DNA complexes should permit kinetic analyses of processes that are difficult to determine in multi-round conditions, such as promoter escape. Titrating all four NTPs in the presence of the DNA trap resulted in an NTP-concentration-dependent increase in signal amplitude, where the traces could be well-fit by a single-exponential function (Supplementary Figure S4B). This NTP-concentration-dependent change in amplitude suggests that the rate of RNAP dissociation from the promoter is on the same order as the rate of promoter escape under these conditions. This observation is consistent with our previous transient-state kinetic measurements of *Mtb* promoter escape kinetics, where we observed that increasing NTP concentration stabilizes the RNAP-DNA complexes, slowing the rate of dissociation and facilitating escape (20). These results illustrate that this fluorescent-aptamer-based assay can be used under single-round conditions to examine the kinetics of sub-steps in initiation (i.e. dissociation and promoter escape).

### Extracting steady-state rates from real-time, multi-round fluorescent traces

**Figure 3.**
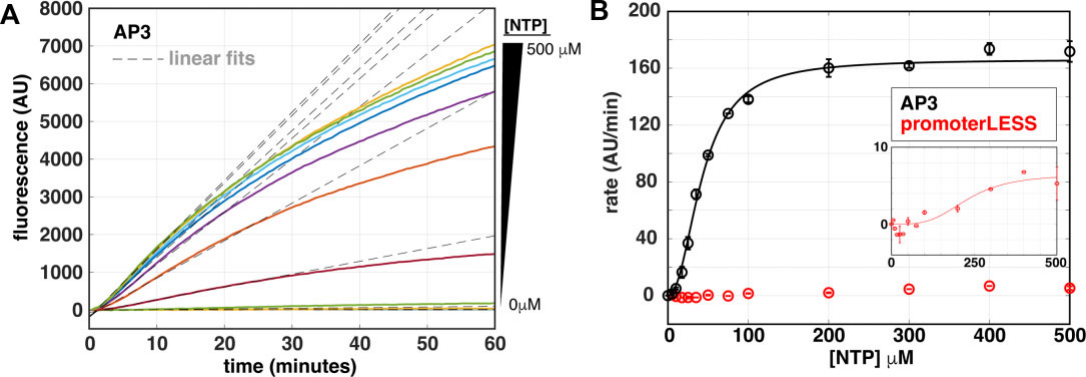
Quantification of the NTP-dependence of steady-state rates for full-length multi-round transcription. (**A**) Titration of the concentration of all NTPs with 100 nM *Mtb* RNAP and 5 nM *rrnA*P3 circular plasmid DNA results in an increase in the rate and amplitude of the fluorescent traces (solid, coloured lines). The unbiased linear fits of the early times are shown in grey dotted lines for each trace. (**B**) Quantification of steady-state rates on *rrnA*P3 (black) and promoterLESS (red) circular plasmid DNA templates plotted as a function of NTP concentration and fit to a modified Michaelis-Menten model (Equation 2) to account for the apparent sigmoidal behaviour.

Under multi-round conditions to examine the steady-state rates of transcription, we observed that the fluorescent time traces exhibited an apparent lag, followed by a linear increase in fluorescence over time. This rate of increase slows over longer timescales and eventually begins to plateau (Figure 3A, solid lines). We hypothesized that saturation of the fluorescence signal can be explained by the presence of paused and/or backtracked polymerases trapped on the template (reviewed in (53)), leading to a reduction in the RNAP molecules that can re-initiate at the promoter over time and an inability of active polymerases to complete transcription of a full length RNA. Consistent with this hypothesis, traces collected with *E. coli* RNAP in the presence and absence of the RNA cleavage factor GreB overlap at early timepoints and then diverged, where conditions containing GreB resulted in a higher fluorescent signal (Supplementary Figure S5). Based on these results, we suggest that GreB acts to increase the active RNAP concentration at longer times by facilitating recycling of long paused/backtracked elongation complexes (54,55). As GreB had no effect in the initial linear region, we suggest that this region reports on the true steady-state rate of initiation, without interference from inactive elongation states occurring downstream of the promoter. In addition, these results suggest that the fluorescent-aptamer-based assay can also be used to examine the regulation of rate-limiting elongation processes at long timescales.

Based on these results, we analyzed the initial fluorescence increase to quantitate the steady-state rate. Previous work manually determined a time regime of the trace to fit with either a line or an exponential. The slope was then determined directly or through differentiation, incorporating an initial time offset to account for the lag-phase (43). To define the steady-state (linear) regime, we designed an automated fitting protocol to reproducibly fit large amounts of data. With this strategy, there is no need to pre-determine either a time range to fit or a functional form representing the entire time course, both of which can change depending on the conditions of individual experiments. First, based on inspection of the traces to be fit, the user defines an initial time for the fitting procedure. In practice, this time should be past any lag or non-linear initial phase in the data. Then, each trace in the input data set are recursively fit with a line, using a statistical weight determined by the variation between technical replicates. The length of the time frame in each round of fitting is reduced by shortening the fit time frame (i.e. decreasing the time of the final data point considered in the fit), and the *R*^2^ value (goodness of fit) is compared to a threshold set by the user to evaluate whether the fit was adequately linear or whether a further reduction in the time frame should be attempted (Supplementary Figure S6). The code then plots the raw data and the fit for visual inspection by the user. Finally, the estimated rates are outputted for subsequent analyses.

### Steady-state kinetic measurements are dependent on NTP concentration

We examined the NTP concentration dependence of reaction rates to ensure that the time-dependent signal change in fluorescence follows our expectations of steady-state behavior. In addition to promoter DNA, NTPs can also be considered as a substrate in the Michaelis-Menten analysis of the transcription reaction. In this case, concentrations below those required to reach *V*_max_ can lead to a decrease in the rate of transcription, generally attributed to slower nucleotide incorporation rates and/or increases in the abortive fraction (14,20). Titrating all four NTPs together up to 500 μM on the *rrnA*P3 circular plasmid template, we observed a clear concentration dependence in the real-time traces (Figure 3A, solid lines). We fit the entire data set with our variable-time fitting algorithm described above, permitting us to extract the steady-state rates at each NTP concentration (Figure 3A, dotted lines). We plotted these rates as a function of NTP concentration and fit the data to a modified Michaelis-Menten equation, weighted by the exponential parameter *n* (Equation 2, Materials and Methods) (Figure 3B). The data did not fit the simpler form of the equation where *n* = 1 and an unconstrained fit results in an *n* = 2.1 ± 0.4. When *n* > 1, it signifies a steeper concentration dependence than expected from Michaelis-Menten equation for a single substrate binding site. Possible interpretations for this non-hyperbolic behavior can be found in the Discussion.

We performed analogous NTP titrations on the promoterLESS circular plasmid template. Compared to the kinetic parameters obtained with the *rrnA*P3 circular plasmid, we observed an ∼25-fold decrease in *V_max_* (6.5 ± 3.8 AU/min compared to 165 ± 7 AU/min) and an ∼5-fold increase in the apparent *K_m_* (*K_m,app_* defined in Methods; 230 ± 120 μM compared to 44 ± 5 μM) (Figure 3B, inset). As mentioned above, given that the signal from the promoterLESS circular plasmid is above that of the no-aptamer control obtained at saturating NTPs (Figure 2A), we interpret the NTP-dependent signal using the promoterLESS template as a measure of the non-*rrnA*P3 promoter derived background transcription. Being able to measure these NTP concentration-dependent trends in signal demonstrates that the assay possesses the detection sensitivity to measure low rates of full-length transcription, including those produced from sequences nominally devoid of promoters. As the promoterLESS control sets a lower bound for detecting *rrnA*P3 promoter-dependent transcription, it should always be included and, if need be, used as a correction. In this specific case, subtraction of the promoterLESS signal from that obtained with *rrnA*P3 resulted in no significant change in Michaelis-Menten parameters *V_max_*, *K_m,app_* or *n* (data not shown). These results indicate that we can confidently assign the fitted kinetic parameters to a *rrnA*P3-derived product under these experimental conditions.

### Steady-state measurements show expected dependence on both RNAP and DNA concentration

In bacterial cells there exists a large molar excess of genomic DNA relative to the amount of free RNAP, making the *in vivo* global rate of transcription largely independent of gene concentration (56–59). Under these conditions, if the free RNAP concentration is well below the *K_m_*, the initial rate becomes independent of DNA concentration and proportional to the free RNAP concentration. As a result, the specific ratios of total RNAP concentration, free RNAP concentration (i.e. RNAPs that are not non-specifically bound to DNA or actively performing transcription (60,61)) and the number of possible interaction sites on the DNA will determine the reaction rate. To illustrate these concepts *in vitro*, we performed DNA titrations at multiple RNAP concentrations, using both circular plasmid (2557 bp) and linear PCR templates (250 bp) to provide different numbers of non-*rrnA*P3 specific interaction sites for the RNAP.

**Figure 4.**
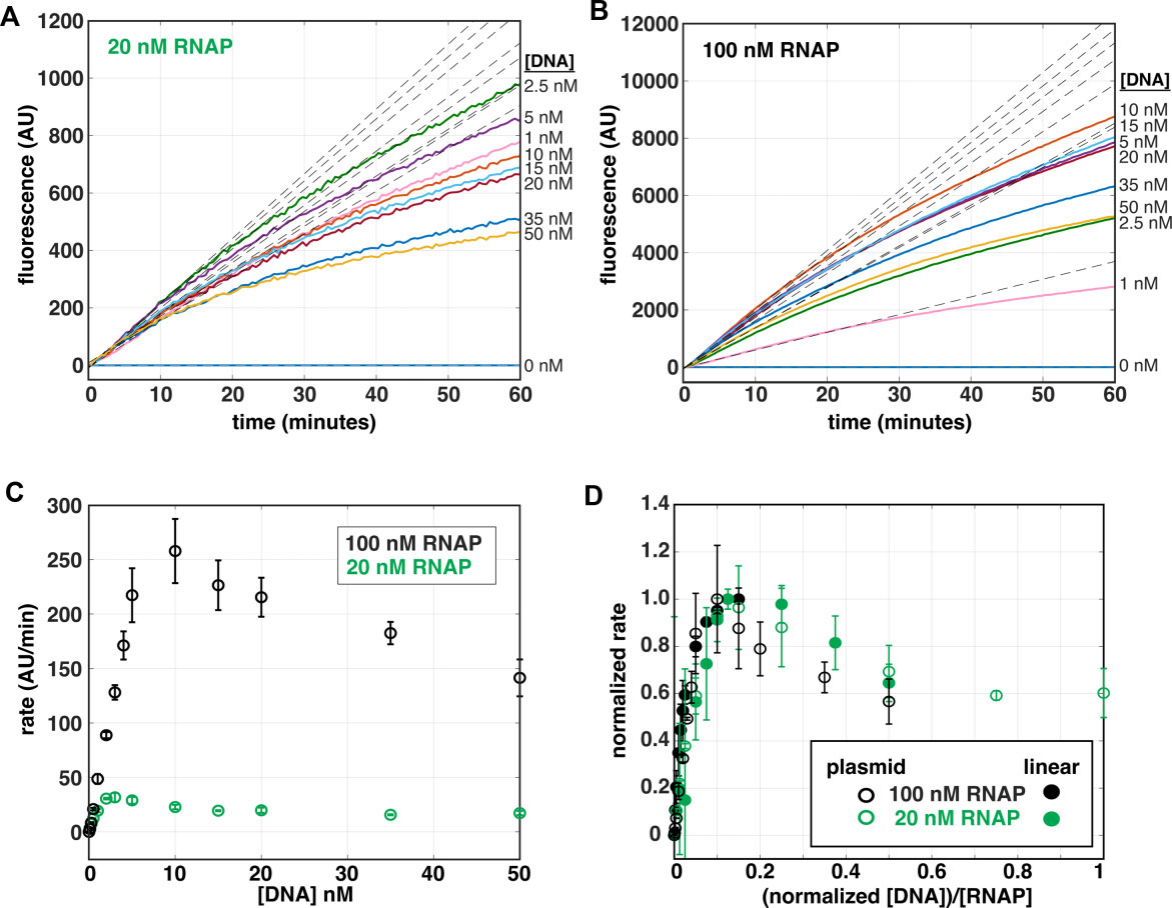
Steady-state rates exhibit a biphasic DNA-concentration-dependence at multiple RNAP concentrations. Real-time data obtained at 500 μM all NTPs, titrating *Mtb rrnA*P3 circular plasmid DNA template (0.1–50 nM) at either **(A)** 20 nM or **(B)** 100 nM *Mtb* RNAP concentrations. The unbiased linear fits of the early times are shown in grey dotted lines for each trace. **(C)** Steady**-**state rates obtained from the linear fits in (A) and (B) for 20 nM (green) and 100 nM (black) RNAP, plotted as a function of *rrnA*P3 circular plasmid DNA concentration. **(D)** Steady-state rates, normalized from zero to one based on the lowest and highest rate obtained at each RNAP concentration, plotted as a function of the normalized [DNA]:[RNAP] concentration ratios. Included titrations of steady-state rate data are those obtained from the circular plasmids templates (open circles) and from linear PCR templates (Supplementary Figure S7, closed circles). Linear PCR DNA template concentration was normalized to that of the plasmid by dividing by a factor of ten to account for the template length (linear PCR template = 250 bp; circular plasmid template = 2557 bp).

Previous measurements using an aptamer-based assay with T7 RNAP illustrated that the DNA concentration dependencies of steady-state rates followed a hyperbolic curve and could be described by Michaelis-Menten kinetics (39). We performed titrations of the *rrnA*P3 circular plasmid (0.1–50 nM) at two different concentrations of *Mtb* RNAP (20 and 100 nM) (Figure 4A, B) and observed significantly higher rates at the higher RNAP concentration (Figure 4C) as expected given the excess of RNAP over DNA. However, we did not observe a monotonic increase in the rates as a function of DNA concentration at either RNAP concentration. At low nM concentrations of the plasmid, we observed the expected increase in steady-state rate; however, as the plasmid concentration was increased, the rate exhibited a concentration-dependent decrease rather than a plateau (Figure 4C). Of note, the peak velocity at both concentrations of RNAP occurred at a similar DNA:RNAP ratio, roughly when RNAP was in 10-fold excess of plasmid DNA (Figure 4D). These observations are consistent with a mechanism where *rrnA*P3 promoter-specific DNA binding dominates in conditions of limiting DNA concentration, but as the concentration of total binding sites is increased, RNAP binds to sites other than *rrnA*P3 more often, resulting in a reduction in specific activity.

Analogous titrations done on linear PCR templates containing the *rrnA*P3 promoter sequence (see Supplementary Table S2 for template preparation, description, and sequence), (Supplementary Figure S7A, B) displayed a less prevalent reduction in steady-state rates at higher DNA concentrations (Supplementary Figure S7C, D). Specifically, the reduction in steady-state rate did not occur until reaching a DNA:RNAP ratio of ∼2:1 for the 20 nM RNAP condition (Supplementary Figure S7D) and no reduction was observed in the data collected with 100 nM RNAP over the DNA concentration range tested (Supplementary Figure S7C,D). Thus, on these short linear PCR templates, the maximal transcriptional activity occurred at a higher DNA:RNAP ratio compared to circular plasmid templates. A shift to a higher ratio of the peak velocity may be caused by a decrease in non-*rrnA*P3 sites. When we normalize the DNA concentration by length, the maximal activities for both linear PCR and circular plasmid templates overlayed, suggesting that the rate decrease is due to non-*rrnA*P3 specific binding (Figure 4D). Combined, these experiments demonstrate quantification of the dependencies of the steady-state rates on both RNAP and DNA concentration, as well as highlight the advantages of using low DNA concentrations when quantifying promoter-specific initiation rates. This is especially important on plasmid templates which typically contain a higher number of possible interaction sites other than the promoter sequence under study.

### Comparisons between fluorescence and gel-based approaches

**Figure 5.**
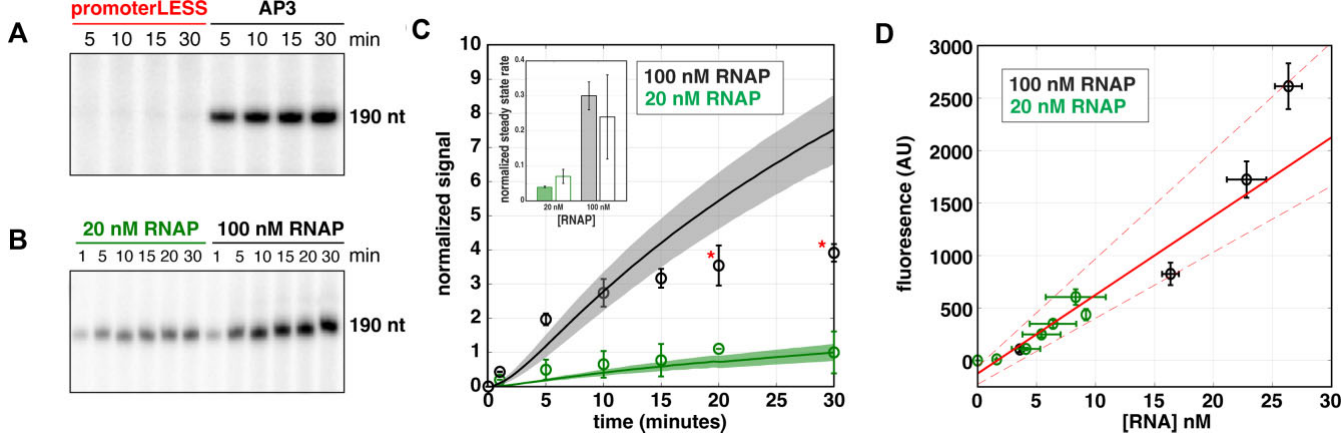
Comparison and calibration of gel- and fluorescence-based kinetics. Transcription gels showing the increase in amount of the specific, full-length ^32^P-labeled transcript with time (see Supplementary Figure S8 for full gel images) obtained with **(A)** 100 nM *Mtb* RNAP and 5 nM of the promoterLESS or *rrnA*P3 circular plasmid templates and **(B)** 20 nM and 100 nM *Mtb* RNAP with 5 nM *rrnA*P3 circular plasmid template. **(C)** Comparison of time-dependent signals from the fluorescent-aptamer-based assay (lines) and the gel assay (open circles) as a function of time for 20 nM and 100 nM RNAP. Both gel data sets were divided by the signal at 30 min obtained with the 20 nM RNAP concentration, thus normalizing that signal to a value of 1. The fluorescent data sets were then normalized using a factor determined by the ratio of the fluorescent and gel data at the same timepoint. The two gel data points that deviate from the fluorescent time course are indicated with red asterisks. The inset shows a comparison of normalized steady state rates from the gel (open bars) and fluorescence (filled bars) data with 20 nM (green) and 100 nM (black) RNAP concentrations. **(D)** Fluorescent signal plotted against RNA concentration from the data shown in (B) excluding the 20 and 30 minute time points obtained with 100 nM RNAP. A linear fit (solid red line) along with 95% confidence regions (dashed red lines) are indicated. The best fit line has a slope of 75 ± 17 AU/nM RNA and an y-intercept of –125 ± 70 AU. Error bars indicate the standard deviations from 3 to 4 independent experiments for fluorescence data and 2–3 independent experiments for gel-based data in all sub-plots.

Our fluorescence measurements clearly exhibit a promoter dependence (Figure 3B). As expected, performing analogous low throughput experiments involving incorporation of ^32^P labeled UTP followed by separation via polyacrylamide gel electrophoresis (Methods) revealed a specific band of the expected length in accordance with the position of the termination sequence on the circular plasmid containing the *rrnA*P3 promoter but not on the promoterLESS circular plasmid template (Figure 5A). In addition, bands larger than the full-length product were observed with both templates (Supplementary Figure S8A). As these bands were of equal intensity in both the *rrnA*P3 and promoterLESS data, we concluded that their generation was due to a component of the plasmid (possibly other cryptic promoters found on the plasmid) other than the promoter region being studied and they are not considered further.

We next asked whether the time evolution of the promoter-specific band yielded the same kinetics as those measured in real-time via the aptamer assay. For this comparison, gel-based experiments were performed at 20 and 100 nM *Mtb* RNAP with 5 nM *rrnA*P3 circular plasmid DNA (Figure 5B; Supplementary Figure S8B). We then normalized both sets of data using the 30-minute timepoint from the 20 nM RNAP condition and compared the time dependences of each signal (Figure 5C). With the exception of the 100 nM RNAP 20- and 30-minute timepoints, the normalized curves overlap within error. Additionally, a comparison of the two approaches shows that the normalized steady-state rates at each RNAP concentration are consistent (Figure 5C, inset). This analysis provides further evidence that the fluorescence signal reports on the promoter-specific activity of the system. Furthermore, it suggests that one may cautiously use the calculated RNA concentration of the specific, promoter-derived band from gel quantifications to convert the arbitrary fluorescence signal into an estimated RNA concentration. Fluorescence signal and RNA concentration were plotted against one another to construct a calibration curve omitting the long time points from the 100 nM RNAP condition (Figure 5D). The best fit line (Figure 5D, red) resulted in a negative y-intercept suggesting that the radioactive assay is more sensitive at low RNA concentrations. In particular, it suggests a lower limit of detection via the aptamer assay of approximately 1 nM, consistent with previous estimates (41). The slope of this calibration was 75 ± 17 AU/nM RNA. We note this conversion factor will not be universal and will depend highly on the experimental setup and conditions (see Supplementary Discussion). In summary, results from the gel-based and fluorescence-based assays are consistent and performing both assays provides a means of converting the arbitrary fluorescence signal into absolute RNA concentration.

### The spinach aptamer and DFHBI have no effect on the measured steady-state rates

We have demonstrated that use of the aptamer assay allows for the quantification of steady-state rates of transcription. However, the two additional elements that could theoretically alter the measured rate have not been discussed: the aptamer sequence and the DFHBI dye. To examine the effect of the aptamer sequence or the presence of the dye on the rate of transcription, we performed ^32^P incorporation assay measurements on circular plasmid templates containing the *rrnA*P3 promoter in the presence and absence of the aptamer sequence and in the presence and absence of dye. We observed roughly the same steady-state rate ± aptamer sequence (Supplementary Figure S9A, B), suggesting that the fluorescence assay can be taken to report on the rates of transcript production without effects from the aptamer. Additionally, in titrating DFHBI concentration up to 20 μM (the concentration used in the fluorescence assay) in ^32^P incorporation experiments, we observed no change in the amount of RNA generated 30 min after initiating the transcription reaction (Supplementary Figure S9C, D), consistent with previous measurements made with T7 RNAP (39,62).

We note that in the aptamer assay, we observe a change in the magnitude of the fluorescent signal when titrating dye concentrations in the context of the same transcription reaction (Supplementary Figure S10A). This can be explained by the finite affinity of the dye for the aptamer. As dye concentration is increased, higher and higher fractions of aptamer are bound, effectively increasing the gain of the signal. However, if experiments are always performed at a fixed dye concentration, preferably higher than the *K*_d_ to maximize signal (Supplementary Figure S10B), comparing fold-changes in fluorescence is a valid approach under steady-state conditions (Supplementary Figure S10C, D). Care should be taken when using large concentrations of dye, as a correction for an inner filter effect may be needed (63).

### High-throughput capabilities of the fluorescent assay permits concentration dependencies of individual NTPs to be measured in a single experiment

**Figure 6.**
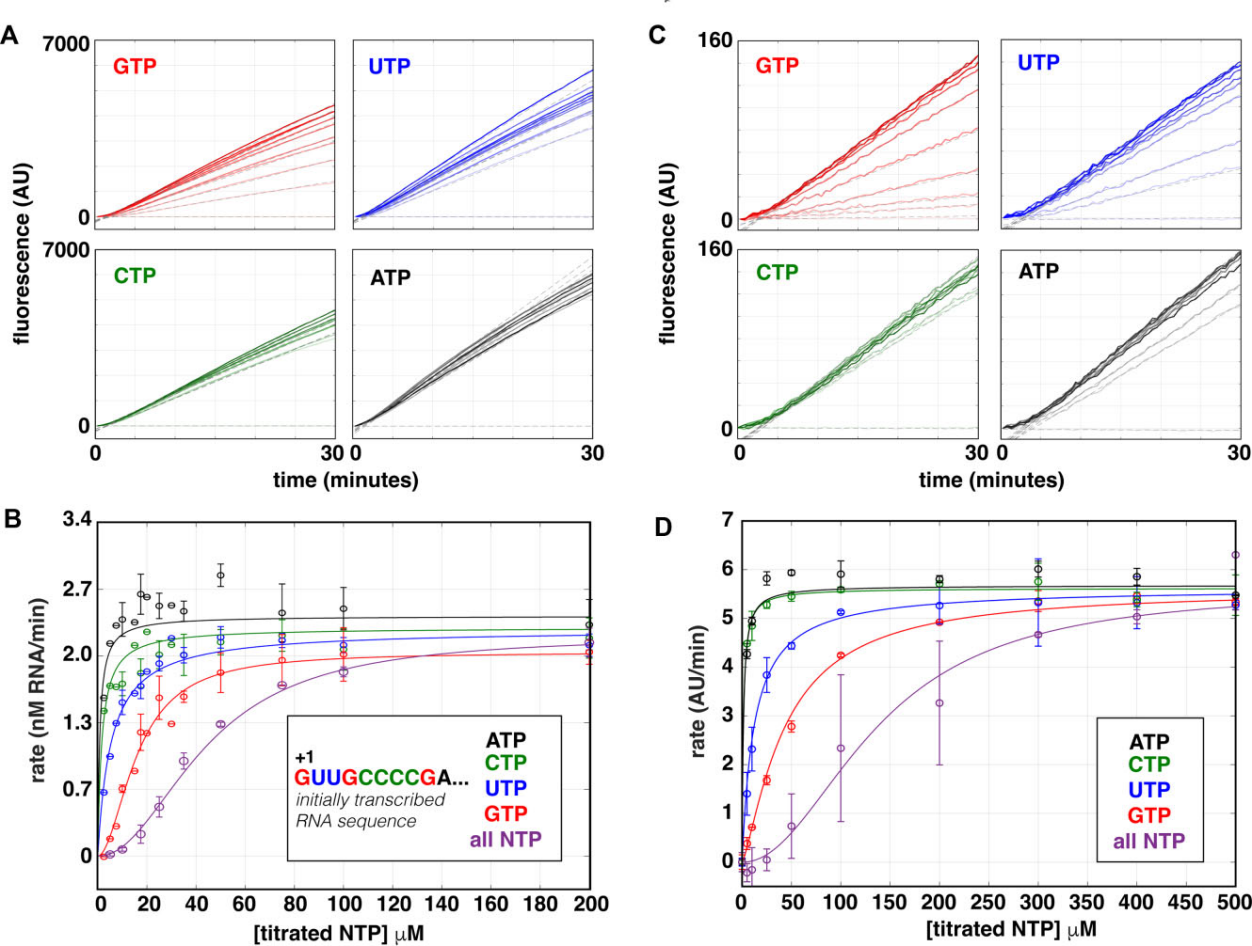
Titrations of individual NTPs reveal incorporation of the initiating nucleotide is rate limiting. **(A)** Real-time fluorescent traces and linear fits obtained using 100 nM *Mtb* RNAP, 5 nM *rrnA*P3 circular plasmid DNA, and individually titrating GTP, UTP, CTP, or ATP in the presence of 500 μM of the other three, non-titrated NTPs. **(B)** Rate dependence on the concentration of individual NTPs from the data in (A) as well as the titration of all NTPs equally. Note that for clarity, only the titration data out to 200 μM NTPs is shown. The y-axis was converted to RNA concentrations using the calibration presented in Figure 5D. **(C)** Real-time fluorescent traces and fits as in (A), except using 25 nM of the *rrnA*P3 linear PCR DNA template. **(D)** Rate dependence on the concentration of individual NTPs from the data in (C) as well as the titration of all NTPs equally. Error bars in (C) and (D) represent standard deviations of 2–4 independent experiments each with 2–3 technical replicates. Rate dependencies in (C) and (D) were fit to Equation 1 or 2 depending on the identity of the titrated NTP, and fitted parameters are summarized in Supplementary Table S3.

Higher concentrations of the initiating nucleotide (iNTP) than the subsequent NTPs are frequently required for promoter-specific initiation, especially on ribosomal RNA promoters where incorporation of the iNTP increases the population of open complexes at equilibrium, which can be a rate-limiting step (20,64). We hypothesized that when measured under steady-state conditions, titrations of the iNTP would thus yield the highest *K_m_* compared to the other NTPs on the *Mtb* ribosomal RNA promoter. To measure the dependence of steady-state rates on the concentration of individual NTPs on the *rrnA*P3 circular plasmid template, we performed titrations of each NTP from 2.5–500 μM in a background of saturating concentrations (500 μM) of the other three NTPs (Figure 6A). Each independent experiment incorporated three technical replicates across ten concentrations per individual NTP titration resulting in a total of 120 total kinetic measurements obtained in parallel, highlighting the high-throughput nature of this assay. The resulting traces were fit to extract steady-state rates as a function of individual NTP concentration (Figure 6B). When the iNTP (GTP) or the second incorporated NTP (UTP) were titrated, the Michaelis-Menten equation (Equation 1) was unable to account for the data. For these titrations Equation 2 was used to account for the sigmoidal dependence. The *V_max_* for each titration was equal within error (Figure 6A,B; Supplementary Table S3). As predicted, the data revealed that the transcription rate depends most strongly on the iNTP (GTP) concentration with a *K_m,app_* of 16 ± 2 μM, consistent with the results from single time-point experiments on *E. coli* ribosomal RNA promoters (64). As the first incorporation site of the titrated nucleotide is found further downstream within the sequence of the initially transcribed region, the measured *K_m,app_* shifted to lower concentrations (Figure 6B; Supplementary Table S3). Notably, these trends in *K_m,app_* and *V_max_* as a function of nucleotide identity were recapitulated using short linear PCR templates containing the *rrnA*P3 sequence (Figure 6C, D). Furthermore, the *K_m,app_* values were either within error or higher in all cases compared to those determined with the circular plasmid template (Figure 6D; Supplementary Table S3), consistent with previous linear and plasmid template comparisons made on ribosomal RNA promoters (64,65). Even though we observed a substantially lower *V_max_* when considering the scale of arbitrary fluorescent units on linear as opposed to plasmid templates, the fact that these nucleotide-identity-dependent trends could be easily measured on either template, including the sigmoidal concentration dependencies, demonstrates the utility of the assay regardless of the type of template used.

### Quantification of transcription factor activity under steady-state conditions

When evaluating the effect of a transcription factor *in vitro*, the most common approach is to use single timepoint measurements to compare transcription in the presence and absence of the factor. However, by only evaluating a single timepoint for each condition there is no guarantee or confirmation that the measurements are representative of changes occurring throughout a steady-state process. In contrast, the aptamer assay directly measures the effect of transcription factors in real-time and by focusing the analysis on the linear regime in an unbiased manner, ensures that factor-dependent changes are quantified under steady-state conditions. To demonstrate the use of the assay in analysis of transcription factor effects, we turned to two well-studied *Mtb* transcription factors, CarD and RbpA.

**Figure 7.**
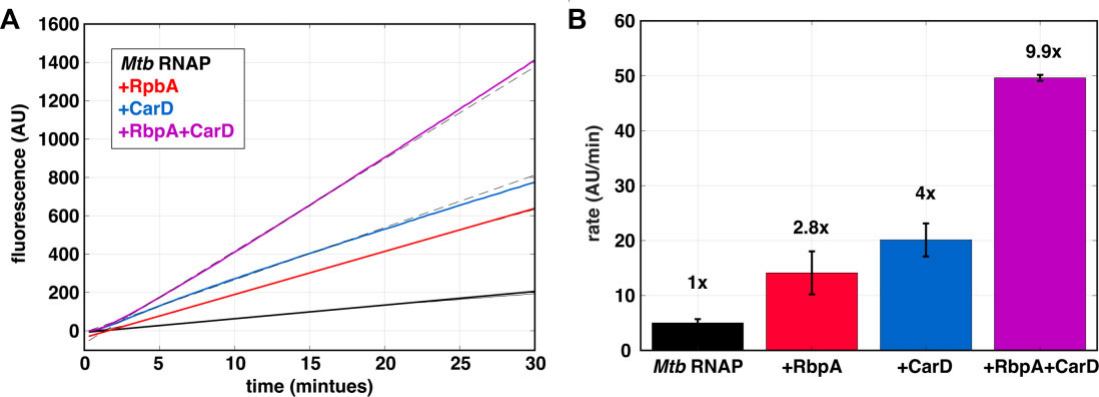
Factor-dependent effects on steady-state transcription rates. **(A)** Real-time fluorescent traces collected by pre-incubating 100 nM *Mtb* RNAP and 500 μM NTPs in the presence of saturating *Mtb* CarD (1 μM) and RbpA (2 μM) (solid curves) prior to addition of 25 nM *rrnA*P3 linear PCR template. Raw traces are shown with associated linear fits (dotted lines). **(B)** Comparison of steady-state rates (average of 2–5 independent experiments) reveals the extent of transcriptional activation by RbpA (red), CarD (blue), and the combination of RbpA and CarD (purple). Fold changes in the steady-state rate relative to *Mtb* RNAP alone are indicated above each condition.

Previous studies have shown that both CarD and RbpA stabilize the open complex (18,19,66,67), slow promoter escape (20) and activate transcription from the *rrnA*P3 promoter (66,68). In addition, these factors are known to bind the initiation complex cooperatively and act synergistically (19,20,67). We measured transcription in the presence of CarD, RbpA, or both at saturating concentrations on the linear PCR *rrnA*P3 template. We monitored fluorescence over time and fit the traces to obtain steady-state rates (Figure 7A). Between 2 and 5 independent experiments were used to calculate and compare the average rates at each condition. Consistent with previous work, we observed a 2.8-fold, 4-fold and 9.9-fold increase in the rate of RNA production in the presence of RbpA, CarD and both factors together, respectively (Figure 7B).

### Quantification of inhibitory concentrations of antibiotics

Bacterial RNAPs are the direct targets of many antibiotics (reviewed in (12,69)) and the search for new antibiotics to overcome drug resistance is never-ending. This is particularly important in the battle against *Mtb*, the causative agent of tuberculosis, as multi-drug resistant strains are becoming more prevalent (70). To this end, the development of new small molecule inhibitors that work at the level of transcription is of high interest. Here, we demonstrate that the aptamer assay can be used to quantify antibiotic-dependent inhibition of steady-state rates. We illustrate this with the well-characterized antibiotics Rifampicin and Fidaxomicin, currently used to treat *Mtb* and *Clostridium difficile* infections by directly targeting the bacterial RNAP (71–74).

**Figure 8.**
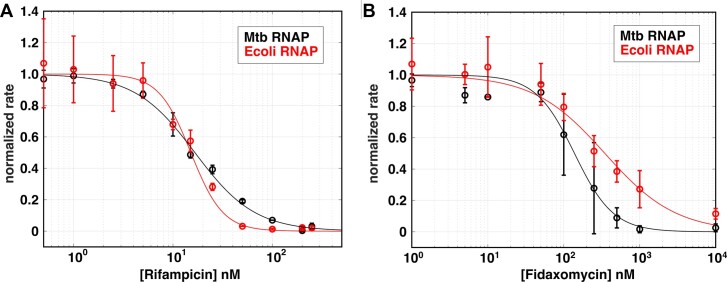
Quantification of antibiotic *IC_50_* values based on changes in observed steady-state rates. Normalized steady-state rates plotted as a function of **(A)** Rifampicin and **(B)** Fidaxomicin concentrations for *Mtb* (black) and *E. coli* (red) RNAP. All experiments were performed using 1 mM of each NTP and 5 nM *rrnA*P3 plasmid DNA. See Supplementary Figure S11 for the corresponding real-time data, linear fits, and un-normalized rates. The normalized steady-state rates are based on the associated fits using Equation 3 (Supplementary Figure S11E, F).

Titrations of both Rifampicin and Fidaxomicin were performed using either 100 nM *Mtb* or *E. coli* RNAP with 5 nM *Mtb rrnA*P3 circular plasmid template (Supplementary Materials and Methods), and the kinetic traces were fit to extract the steady-state rates. Although *E. coli* RNAP exhibits a lower maximal rate of transcription on this template, as inferred from the fluorescence signal in the absence of antibiotic (Supplementary Figure S11), for both the RNAPs, we observed a concentration-dependent decrease in the measured steady-state rates upon increasing antibiotic concentrations (Figure 8). Fitting the data to an inhibition curve (Equation 3) permitted the calculation of half-maximal inhibitory concentrations. Consistent with previous work, the *IC_50_* for Rifampicin on *E. coli* RNAP (15 ± 2 nM) and *Mtb* RNAP (17 ± 2 nM) were within error (Figure 8A) (66,75). In addition, the same analysis for Fidaxomicin yielded an approximately three-fold higher *IC_50_* on *E. coli* RNAP (400 ± 110 nM) than on *Mtb* RNAP (138 ± 92 nM) (Figure 8B), consistent with previous reports of *Mtb* being more sensitive to the drug than *E. coli* (72,76).

## DISCUSSION

### High-throughput kinetic measurements of full-length transcription

The fluorescence experiments presented here are an attractive alternative technique to the standard radiolabeled-nucleotide incorporated gel-based approaches when used to quantitate steady-state transcription kinetics, especially when used in a plate-reader format as described. Hundreds of conditions can be measured in real-time with several technical replicates in a single experiment. We estimate that the throughput is on the order of a hundred times that of standard gel-based approaches, where the real-time measurements facilitate a more precise and accurate determination and quantification of the steady-state regime. As the steady-state rate of full-length transcription (along with the RNA degradation rate) is a crucial metric when considering the flux of biological processes that use RNA as a substrate (1), use of this assay will significantly facilitate the exploration of diverse mechanisms of gene regulation with biological relevance.

We emphasize that radioactive techniques are advantageous and complementary when looking to resolve individual RNA transcripts, as well as to determine the relative lengths and amounts of side and abortive products (77). In fact, the first steady-state assays that reported on bacterial transcription were made under conditions where NTP synthesis was restricted to abortive transcription (78,79). However, as RNAP does not escape the promoter under these circumstances, these assays did not report on the overall rate of initiation, but rather the rate-limiting step involved in binding and isomerization to the catalytically active open complex (79). Fluorescent-labeled nucleotides that are incorporated into the RNA transcripts have also been used to monitor the steady-state rates of abortive RNA generation with *E. coli* RNAP (80–83), representing an alternative to radioactivity-based approaches. However, this method cannot discriminate between abortive and full-length RNA products. In comparison to other techniques, the fluorescent aptamer-based assay described here is advantageous, as the observed fluorescence signal reports specifically on full-length RNA products. This is demonstrated in conditions where single NTPs are not included, resulting in the inability of RNAP to escape the promoter and a lack of fluorescence signal change (Figure 6). We note that the effects of abortive synthesis are still borne out in the kinetics, as the process of promoter escape can be rate-limiting on some promoters and affected by the initially transcribed sequence (84,85), which we have included in our DNA constructs. As a result, the assay permits a straightforward way to measure the rate of full-length transcript production dictated by the overall rate of initiation.

We have illustrated that the assay can easily quantitate differences in kinetics due to promoter sequence (Figure 3), NTP concentration (Figure 6), and RNAP and promoter DNA concentrations (Figure 4; Supplementary Figure S7). The non-monotonic rate dependencies observed when titrating DNA (Figure 4; Supplementary Figure S7) suggest one could use this assay to evaluate how the presence of other promoters or number of non-promoter sites compete for RNAP binding and affect the steady-states rates from a particular promoter of interest. Furthermore, in addition to having great utility in multi-round assays, the assay can be used in single-round conditions to evaluate rates of initiation processes such as promoter escape (Supplementary Figure S4). The single-round approach has previously been well-described and used for monitoring co-transcriptional RNA folding processes (43,51).

We stress that meaningful quantifications of relative changes in flux can be made using arbitrary fluorescence units as long as buffer conditions, temperature, or any other variable that may affect aptamer folding or DFHBI binding are not varied. However, we have also shown the approach and necessary controls to calibrate the fluorescent signal using an independent measure of RNA concentration from identical reactions (Figure 5). Further thoughts on calibration and interpretation of the fluorescent signal can be found in the Supplementary Discussion.

### Nucleotide-dependent kinetics

By performing titrations of NTP substrates, we have illustrated that *K_m,app_* and *V_max_* parameters can be obtained with a high level of precision under steady-state conditions (Figure 6). Using these data, we observed that not all titrations could be fit to a hyperbolic Michaelis-Menten model. Rather, sigmoidal trends were observed for both circular plasmid and linear PCR DNA templates in the titration of all NTPs together and GTP in the background of saturating concentrations of ATP, UTP, and CTP (Figure 6B, D; Supplementary Table S3). Initiation requires the first two nucleotides to form the first RNA phosphodiester bond and, depending on the conformation of the open DNA, base-stacking with the +1/+2 DNA sequences on the template strand may represent a rate-limiting step (17). Our results are consistent with this hypothesis in that we observe that the concentration of the first two NTP substrates had a larger effect as compared to the other NTPs (Figure 6B, D; Supplementary Table S3). For monomeric enzymes containing a single active site, a sigmoidal dependence of velocity on substrate concentration has been referred to as kinetic cooperativity and was first measured with glucokinase (86,87). Additionally, other mechanisms have been suggested to lead to kinetic cooperativity without direct interactions between binding sites (reviewed in (49)), including the existence of a slow conformational change that precedes substrate incorporation, the existence of multiple enzyme classes capable of binding initiating nucleotides with different affinities, and/or a substrate-induced conformational change to the catalytically active enzyme (49,88). Mechanistic explanations may include effects of the first two nucleotides on the ratio of abortive and productive synthesis or the necessity to bind two NTP substrates to synthesize the first phosphodiester bond. These models may not be mutually exclusive and, in fact, are supported by numerous experimental studies evaluating the initial nucleotide incorporation process with *E. coli* RNAP (13,14,16,17). While we cannot directly comment on the specific mechanism(s) that results in this apparent positive cooperativity with *Mtb* RNAP, the detection of this sigmoidal behavior would not have been possible without the fine sampling of NTP concentrations and precise measurements of steady-state rates facilitated by the ease and throughput of the aptamer-based assay.

### Future uses for high-throughput detection of basal and regulated transcription kinetics

Given these fluorescent experiments were all performed in a 384 well plate, the assay lends itself naturally to questions that require large data sets. We conclude by summarizing a few exciting possible directions.

Using sequence to predict transcriptional activity remains a challenge, as the context of the entire promoter must be considered when examining how sequence affects initiation kinetics (3). Using the aptamer-based assay, one could generate promoter libraries where sequence context is investigated by introducing sequence mutations in promoter regions of interest (44,84,89). Alternatively, one could design and measure basal and regulated transcription rates on large numbers of genomic promoter sequences. This would allow for a side-by-side comparison with genome-wide, RNA-seq data (90). Use of the aptamer-based assay will greatly facilitate the ability to quantitatively probe the kinetics of larger genomic promoter libraries *in vitro* to expand our knowledge of gene regulatory mechanisms.

As we illustrated the utility of the fluorescence assay in measuring antibiotic inhibition (Figure 8; Supplementary Figure S11), the high-throughput screening of novel inhibitors against the bacterial transcription machinery may also benefit from this assay. Inhibitors that act at the level of initiation could be identified by a reduction of the initial steady-state rate, as observed for Rifampicin and Fidaxomicin (Figure 8; Supplementary Figure S11). In addition, inhibitors of elongation or termination that lead to stalled, template bound RNAPs may result in changes in the kinetics of the signal over longer-timescales as they eventually reduce the number of actively transcribing polymerases and may become roadblocks to RNAPs that are still active. As a result, use of this assay under multi-round conditions may permit the identification of compounds that inhibit transcription via different mechanisms. Furthermore, because one tracks the time-dependent changes in fluorescence and not single-timepoints, compounds that exhibit fluorescent spectral properties are less likely to produce artifactual results. Compounds identified as potential hits can then easily be analyzed via titrations to measure the *IC_50_*s as described here. To develop structural hypotheses for the mechanism of transcription initiation inhibitors (or classes of inhibitors), titrations of NTPs or promoter DNA could be performed to determine the type of inhibition (e.g. competitive, non-competitive, or uncompetitive) via quantification of changes in *K_m,app_* and/or *V_max_* (91).

## Supplementary Material

gkad761_Supplemental_File

## Data Availability

The MATLAB code used for automated linear regression analyses has been made publicly available and can be accessed via the following link: https://doi.org/10.5281/zenodo.8319359.
