# Supplementary material for: High-throughput, fluorescent-aptamer-based measurements of steady-state transcription rates for the Mycobacterium tuberculosis RNA polymerase
Source: Nucleic Acids Res. 2023 Sep 22;51(19):e99. doi: 10.1093/nar/gkad761 (PMC10602862; doi:10.1093/nar/gkad761)
Supplement: gkad761_Supplemental_File [file gkad761_supplemental_file.docx]

**Supplementary Information for:**

**High-throughput, fluorescent-aptamer-based measurements of steady-state transcription rates for the *Mycobacterium tuberculosis* RNA polymerase**

Drake Jensen^1,†^, Ana Ruiz Manzano^1,†^, Maxwell Rector^2^, Eric J. Tomko^1^, M. Thomas Record^2^, and Eric A. Galburt^1,^*

1. Department of Biochemistry and Molecular Biophysics, Washington University School of Medicine, Saint Louis, MO, USA, 63108
2. Department of Biochemistry, University of Wisconsin, Madison, WI, USA, 53706

* To whom correspondence should be addressed. Tel: +1 314-362-4821; Fax: +1 314-362-7183; Email: egalburt@wustl.edu

^†^ Joint first-authors

**This PDF file includes:**

# Supplementary Materials and Methods

# Supplementary Discussion

# Supplementary Tables

**Table S1:** Insert sequences for circular plasmid DNA templates

**Table S2:** Primer and final sequences for linear PCR DNA templates

**Table S3:** Calculated *K_m_*,*_app_* *V_max_* and *n* values from NTP titrations

# Supplementary Figures

**Figure S1:** Agarose gel of plasmid DNA templates

**Figure S2:** Simulations where aptamer-folding is rate-limiting

**Figure S3:** Real-time fluorescent signal in the absence of aptamer formation

**Figure S4:** Single-round trap controls and kinetics

**Figure S5:** GreB affects the long-term behaviour of fluorescent traces

**Figure S6:** Overview of variable-time, iterative linear fitting approach

**Figure S7:** Real-time data for DNA titrations on linear PCR templates

**Figure S8:** Full gel images of the data presented in main text Figure 5

**Figure S9:** Gel-based analysis of the effect of the aptamer sequence and DFHBI dye

**Figure S10:** Concentration dependencies of DFHBI on the fluorescent-aptamer signal

**Figure S11:** Real-time data for antibiotic titrations and unnormalized *IC_50_* fits

**Supplementary References**

# Supplementary Materials and Methods

**Plate-reader, multi-round experimental conditions**

Reaction conditions are summarized below:

***NTP titrations:*** 100 nM *Mtb* σ^A^ RNAP holoenzyme was pre-incubated with NTP concentrations ranging from 2.5 – 500 μM and the reaction was initiated with either 5 nM *Mtb* *rrnA*P3 or promoterLESS circular plasmid DNA, or 25 nM *Mtb* *rrnA*P3 linear PCR DNA. This 25 nM concentration for linear PCR DNA templates was chosen as it represents the *K_m_* obtained with DNA titrations at this RNAP concentration (**Supplementary Figure 7C**). Titrations with an individual NTP were done in the presence of 500 μM of the other three NTPs. For titrations of all NTPs, the concentrations of each NTP were varied equally.

***DNA titrations:*** 500 μM all NTPs were pre-incubated with either 20 or 100 nM *Mtb* σ^A^ RNAP holoenzyme. The reaction was initiated with concentrations of 0.1 – 50 nM *Mtb* *rrnA*P3 circular plasmid DNA or 2.5 – 150 nM *Mtb* *rrnA*P3 linear PCR DNA.

***DFHBI titrations:*** DFHBI dye (concentrations ranging from 0.005 – 40 μM) was pre-incubated with 100 nM *Mtb* σ^A^ RNAP holoenzyme and 500 μM all NTPs. The reaction initiated with 5 nM *Mtb* *rrnA*P3 circular plasmid DNA. For each dye concentration examined, a negative control (no DNA) was collected to correct for any time-dependent changes in the background fluorescent signal.

***Antibiotic titrations:*** Rifampicin (concentrations ranging from 0.5 – 250 nM), or Fidaxomicin (concentrations ranging from 0.0001 – 100 μM) was preincubated with 100 nM *Mtb* σ^A^ or *E. coli* σ^70^ RNAP holoenzyme and 1 mM all NTPs. The reaction initiated with 5 nM *Mtb* *rrnA*P3 circular plasmid DNA. The final DMSO concentration was kept under 0.2% (v/v) to avoid any concentration-dependent effects of DMSO on RNA folding (1), which would affect the fluorescent signal.

***Transcription Factors:*** Experiments were performed using saturating concentrations of transcription factors, pre-incubated with 100 nM *E. coli* σ^70^ or *Mtb* σ^A^ RNAP holoenzyme and 500 μM all NTPs. Reactions were initiated with 5 nM *Mtb* *rrnA*P3 circular plasmid DNA (*E. coli* GreB) or 25 nM *Mtb* *rrnA*P3 linear PCR DNA (*Mtb* CarD and RbpA). GreB experiments used 1 μM, which binds *E. coli* RNAP core and σ^70^ holoenzyme with ~ 10 nM affinity (2) and elongation complexes with ~ 100 nM affinity (3, 4). CarD and RbpA experiments used 1 and 2 μΜ, respectively, as these concentrations lead to saturable open-complex formation kinetics with the *Mtb* RNAP σ^A^ holoenzyme (5, 6).

## Plate-reader, single-round experimental conditions

To prevent multiple rounds of RNA generation derived from dissociated/terminated RNAPs rebinding the promoter, a competitor DNA was added. We used either Salmon-sperm DNA (Invitrogen; catalogue # 15632011) or a circular plasmid DNA template that contained the *Mtb rrnA*P3 sequence but lacked the aptamer sequence (**Supplementary Table 1**). Pre-formed promoter-bound complexes (100 nM *Mtb* σ^A^ RNAP holoenzyme and 5 nM *Mtb* *rrnA*P3 circular plasmid DNA) with 20 μM DFHBI and 0.4 U/μL RNase inhibitors were initiated with either 75 µg/mL of salmon-sperm DNA or 150 nM “without aptamer” circular plasmid DNA along with all NTPs at various concentrations. These concentrations of competitors when pre-incubated with RNAP and *Mtb* *rrnA*P3 circular plasmid DNA containing the aptamer sequence caused no change in fluorescence upon initiating the reaction with NTPs (**Supplementary Figure 4A**).

## Simulations of steady-state kinetics

Master-equation based simulations were used to explore the effects of aptamer folding and dye binding rates on the observed steady-state fluorescent signal in **Supplementary Figures 2** and **10C,D**. The kinetic model consisted of rate constants describing RNAP-promoter binding (*k_on_*) and dissociation (*k_off_*), promoter escape (*k_escape_*), aptamer folding (*k_folding_*), and dye binding (*k_bind_*) and dissociation (*k_diss_*) and is shown graphically in **Supplementary Figure 2**. Pseudo first order can be assumed for the binding rate constants for RNAP-DNA (*k_on_*) and dye-RNA (*k_bind_*), when these reactants are in excess. These rate constants are proportional to concentrations of RNAP and dye, respectively. All other rate constants are uni-molecular and concentration independent. The simulation begins with 100% of the population in the unbound polymerase state (R+P). The promoter escape step regenerates free polymerase and promoter and produces an unfolded RNA transcript. Thus, the polymerase/promoter system can generate multiple transcripts over time and the population of unfolded transcript can increase without limit. The accumulation of dye-bound, folded aptamer is what is plotted as a function of time and is taken as a measure of the expected fluorescent signal in the actual assay. At steady state, the concentrations of R+P, RP, and unfolded aptamer are constant while the concentrations of unbound and bound folded aptamer increase linearly in a constant ratio dictated by the dye affinity and concentration.

# Supplementary Discussion

## Relative vs. absolute RNA concentration measurements: cautions in calibration and interpretation of florescent signal across different experimental conditions

Throughout this work, we have illustrated that quantifications of relative changes between conditions can be made using arbitrary fluorescence units. However, we have also shown that the fluorescent signal may be calibrated using an independent measure of RNA concentration from identical reactions. The calibration presented here follows previously described methodologies (7–9), and uses the signal intensity of the full-length transcript band resolved from gels (**Figure 5B**) to convert RNA concentration using a standard curve (**Figure 5D**). This approach depends on identification of a promoter-specific transcript of the correct length (**Figure 5A**; **Supplementary Figure 8A**). We note that if the total incorporated radioactivity signal were to be used, an overestimate of the promoter-derived, aptamer containing RNA concentration would result, as can be seen by the presence of non-*rrnA*P3-derived transcripts (**Supplementary Figure 8A**). Similarly, one would also likely overestimate the RNA aptamer concentration if measuring RNA concentration via spectroscopic approaches, such as monitoring absorbance at 260 nm following DNase treatment or monitoring the fluorescence of RNA-specific interacting dyes (7), as both these approaches would measure the total RNA concentration and not just the promoter-derived product containing the aptamer.

Other studies have bypassed gel-based approaches completely and suggested to calibrate RNA amounts by comparing the fluorescent signal of the experiment to that of known concentrations of purified aptamer (10). While attractive in theory due to its simplicity, this assumes that the equilibrium fraction of folded aptamer when purified is the same when co-transcriptionally folded and generated *in situ*. Here, one must use caution as the specific method used for *in vitro* folding can affect the equilibrium amount of folded aptamer that is capable of binding dye and as a result dictate the fluorescent signal (11). In fact, previous calibration attempts confirmed this assumption, where the calibration based on aptamer fluorescence was roughly half that of what was measured based on gel band intensity (8).

We emphasize that calibration values are specific to the aptamer and solution conditions used for the experiment and can’t be applied universally. In fact, even drawing conclusions from relative comparisons when aptamer or solution conditions are modified is not advised since they may alter the relationship between the concentration of RNA and the arbitrary units of fluorescence. Many other fluorescent aptamer sequences have been described and designed, improving on the original Spinach and Spinach-mini sequence used here, by increasing thermostability, folding propensity, quantum-yields, etc. (11–17). As a result, the same amount of transcript generated containing a different aptamer sequence, will invariably yield a different fluorescent signal. Additionally, testing aptamer sequences via gel assays can ensure the sequence has no effect on transcription kinetics **(Supplementary Figure 9)**.

Similarly, care must be taken if one is interested in the effects of solution conditions on transcription. For instance, salt type and concentration can have large effects on both transcription kinetics (18–20) and aptamer folding/dye binding (15, 21, 22). As a result, without performing a calibration curve at each unique salt concentration tested, one could not comment on whether the change in fluorescence is due to transcriptional activity, aptamer folding, or some combination of the two. For this reason, we strongly suggest keeping conditions that affect RNA folding (salt, Mg^2+^, pH, temperature, DMSO, etc.) constant when comparing effects of transcription factors or promoter sequence, or when performing a titration series. For instance, if titrating a component stored in a different buffer than that of the reaction buffer, use the same volume percentage of the component and its storage buffer for each condition, or dialyze all reaction components into the same buffer.

# Supplementary Tables

**Table S1: Insert sequences for circular plasmid DNA templates.** The non-template strand sequences displayed below are the requested inserts that are cloned into the pTwist, High Copy, Amp^R^ plasmid vector (Twist Bioscience). Sequence numbering is relative to the transcription start site (blue, numbered as +1,) where the genomic locations are provided based on the *Mtb* H37Rv numbering**.** The terminator corresponds to 80 nt of the *E. coli* *rrnB*P1 *T*_1_ genomic sequence (red, 6608 to 6687 based on numbering in Ref. (23)). The aptamer corresponds to the Spinach-mini RNA sequence (green) (24). Non-genomic-derived sequences were included as Forward (F-) and Reverse (R-) primer annealing sites (yellow) and can be used to amplify the sequence insert from the plasmid to make linear templates. The linear templates used in this work were prepared in accordance with the methods described in **Supplementary Table 2**.

|  | 1. *Mtb* *rrnA*P3 |
| --- | --- |
| **Description** | F-primer *rrnA*P3 aptamer +39bp *rrnA*P3 +80bp terminator R-primer |
| **DNA Sequence**  **(Non-Template)**  (5’-3’) | GAGCTCGGTACCCGGGGATCATCTATGGATGACCGAACCTGGTCTTGACTCCATTGCCGGATTTGTA  TTAGACTGGCAGGGTTGCCCCGAAGCGGGCGGAAACAAGCAAGCGACGCGACCGAAATGGTGAAG  GACGGGTCCAGTGCTTCGGCACTGTTGAGTAGAGTGTGAGCTCCGTAACTGGTCGCGTCGTGTTGTT  TGAGAACTCAATAGTGTGTTTGGTGGTTTCACCAGGCATCAAATAAAACGAAAGGCTCAGTCGAAAGA  CTGGGCCTTTCGTTTTATCTGTTGTTTGTCGGTGAACGCTCTCGCCTCTCCCCGCGCGTTGGC |
| **Genomic Location** | 1,471,597 (-60) to 1,471,687 (+31); 1,471,688(+32) to 1,471,726(+70) |
|  | 2. *Mtb* *rrnA*P3 without aptamer |
| **Description** | F-primer *rrnA*P3 +80bp terminator R-primer random |
| **DNA Sequence**  **(Non-Template)**  (5’-3’) | GAGCTCGGTACCCGGGGATCATCTATGGATGACCGAACCTGGTCTTGACTCCATTGCCGGATTTGTA  TTAGACTGGCAGGGTTGCCCCGAAGCGGGCGGAAACAAGCAAGCGTGTTGTTTGAGAACTCAATAG  TGTGTTTGGTGGTTTCACCAGGCATCAAATAAAACGAAAGGCTCAGTCGAAAGACTGGGCCTTTCGTT  TTATCTGTTGTTTGTCGGTGAACGCTCTCGCCTCTCCCCGCGCGTTGGCCCCGTCACCAGATTAAGC GGAAAGCGCAAAGCAAGGATTGGGCGTTCCGC |
| **Genomic Location** | 1,471,597 (-60) to 1,471,726(+70) |
|  | 3. PromoterLESS |
| **Description** | F-primer *rrnA*P3 (Δ -49 to -1 of *rrnA*P3) *rrnA*P3 aptamer +39bp *rrnA*P3 +80bp terminator R-primer |
| **DNA Sequence**  **(Non-Template)**  (5’-3’) | GAGCTCGGTACCCGGGGATCATCTATGGATCATGTTTGACAGCTTATCATCGGAGCTCTCGAGTCTA  GAATCGATCCCCGGTTGCCCCGAAGCGGGCGGAAACAAGCAAGCGACGCGACCGAAATGGTGAAG  GACGGGTCCAGTGCTTCGGCACTGTTGAGTAGAGTGTGAGCTCCGTAACTGGTCGCGTCGTGTTGTT  TGAGAACTCAATAGTGTGTTTGGTGGTTTCACCAGGCATCAAATAAAACGAAAGGCTCAGTCGAAAGA  CTGGGCCTTTCGTTTTATCTGTTGTTTGTCGGTGAACGCTCTCGCCTCTCCCCGCGCGTTGGC |
| **Genomic Location** | 1,471,597 (-60) to 1,471,606 (-50); 1,471,657 (+1) to 1,471,687 (+31); 1,471,688(+32) to 1,471,726(+70) |

## *Descriptions*

1. ***Mtb* *rrnA*P3**: Primary template used in this work. Contains both the Spinach-mini aptamer and the *E. coli* *rrnB*P1 *T*_1_ terminator with *Mtb rrnA*P3 genomic sequence (purple) present both upstream (from –60 to +31) and downstream (from +32 to +70) of the aptamer.
2. ***Mtb* *rrnA*P3 without aptamer**: Same design as construct #1, except the aptamer sequence was removed. The randomized sequence (cyan) added after the terminator and R-primer was needed to reach the required minimal insert length.
3. **“PromoterLESS”:** Same design as construct #1 except that –49 to –1 of the *Mtb* *rrnA*P3 genomic DNA was replaced with a randomized sequence (orange).

**Table S2: Primer and final sequences for linear PCR DNA templates.** Note that the genomic sequence numbering and colour coding below follows the same format as described in **Supplementary Table 1**. Primer oligos (sequences shown under blue headings) contained 150 bp of overlapping sequence (annealing region, underlined), leaving 50 nt overhangs on each end. An overview of the preparation steps is included: Annealing and end-filling to generate a 250 bp construct (final non-template sequence shown under grey heading) was accomplished with Platinum Taq DNA Polymerase (Invitrogen; catalogue # 15966005). Once extended, the genomic *rrnA*P3 sequence from –60 to +31 is present upstream of the aptamer and from +32 to +70 is present downstream of the aptamer, as in the DNA plasmid constructs. These templates underwent an additional PCR step (using primer sequences shown in yellow) with Klentaq LA DNA Polymerase (25) (DNA Polymerase Technology; catalogue # 110). We added a biotin molecule to the Forward, 20 nt primer, attached to the 5’-end via a standard C6 spacer (code for modification is /5Biosg/ when ordering from Integrated DNA Technologies, Inc.). The biotin addition was in hopes to reduce end-binding/initiation effects. Following each PCR step, samples were purified with QIAquick PCR Purification Kit (Qiagen; catalogue # 28104) and purity was verified by Native-PAGE.

| **Overview of preparing 250 bp linear constructs** | | | | |
| --- | --- | --- | --- | --- |
| 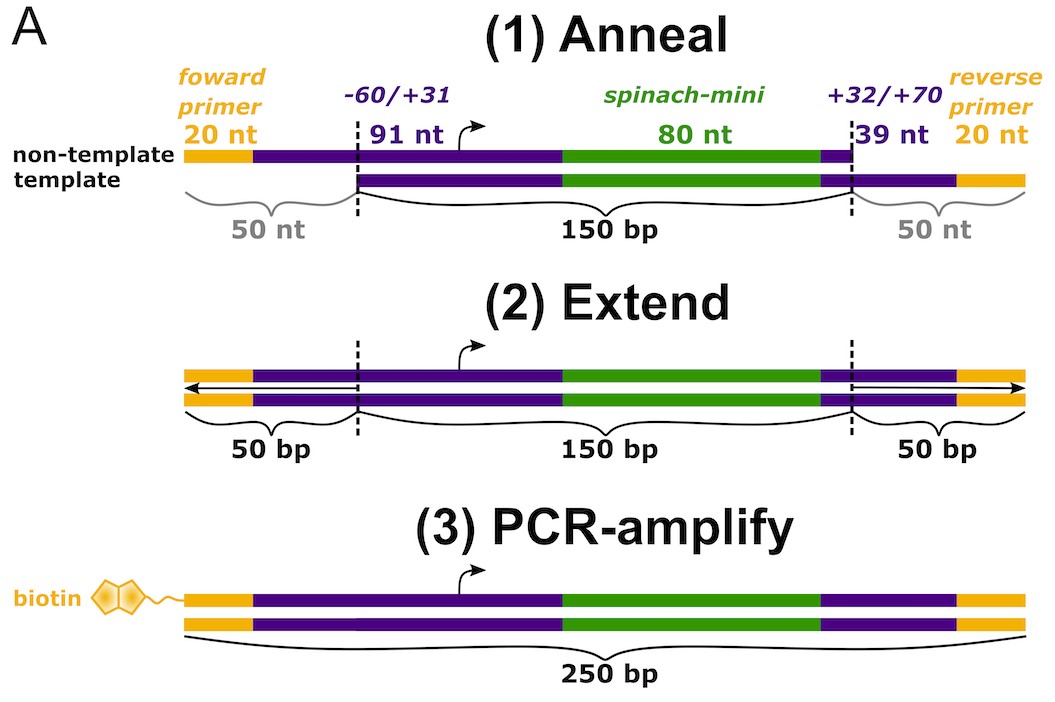 | | | | |
| **Primers for annealing/extending** | | | | |
| **DNA Sequence (5’-3’)** | | **Genomic Location** | **Strand** | **Length**  **(nt)** |
| GAGCTCGGTACCCGGGGATCATCTATGGATGACCGAACCTGGTCTTGACT  CCATTGCCGGATTTGTATTAGACTGGCAGGGTTGCCCCGAAGCGGGCGGA  AACAAGCAAGCGACGCGACCGAAATGGTGAAGGACGGGTCCAGTGCTTCG  GCACTGTTGAGTAGAGTGTGAGCTCCGTAACTGGTCGCGTCGTGTTGTTT | | 1,471,597 (-60) to 1,471,687 (+31);  1,471,688 (+32) to  1,471,696 (+41) | Non-template | 200 |
| GCCAACGCGCGGGGAGAGGCTGAAACCACCAAACACACTATTGAGTTCTC  AAACAACACGACGCGACCAGTTACGGAGCTCACACTCTACTCAACAGTGCC  GAAGCACTGGACCCGTCCTTCACCATTTCGGTCGCGTCGCTTGCTTGTTTC  CGCCCGCTTCGGGGCAACCCTGCCAGTCTAATACAAATCCGGCAATGG | | 1,471,726 (+70) to 1,471,688 (+32);  1,471,687 (+31) to  1,471,627 (-30) | template | 200 |
| **Final non-template sequence** | | | | |
| **Description** | **F-primer *rrnA*P3 aptamer +39bp *rrnA*P3 R-primer** | | | |
| **DNA Sequence**  **(Non-Template)**  (5’-3’) | GAGCTCGGTACCCGGGGATCATCTATGGATGACCGAACCTGGTCTTGACTCCATTGCCGGATTT  GTATTAGACTGGCAGGGTTGCCCCGAAGCGGGCGGAAACAAGCAAGCGACGCGACCGAAATG  GTGAAGGACGGGTCCAGTGCTTCGGCACTGTTGAGTAGAGTGTGAGCTCCGTAACTGGTCGCG  TCGTGTTGTTTGAGAACTCAATAGTGTGTTTGGTGGTTTCAGCCTCTCCCCGCGCGTTGGC | | | |
| **Genomic Location** | 1,471,597(-60) to 1,471,687(+31); 1,471,688(+32) to 1,471,726(+70) | | | |

**Table S3: Calculated *K_m_*,*_app_*, *V_max_*, and *n* values from NTP titrations.** Values and errors provided are from fits to the averaged data. For titrations in the context of circular plasmid DNA templates, RNA concentration was calculated using the calibration factor presented in **Figure 5D**. All NTP titrations were performed with 100 nM *Mtb* RNAP and either 5 nM circular plasmid or 25 nM linear PCR *Mtb* *rrnA*P3 DNA.

|  | **Circular Plasmid DNA** |  | | **Linear PCR DNA** |  | |
| --- | --- | --- | --- | --- | --- | --- |
| **Titrated NTP** | ***Km,app*** (μΜ) | ***Vmax***  (nM RNA/min) | ***n*** | ***Km,app***  (μΜ) | ***Vmax***  (AU/min) | ***n*** |
| **ALL** | 44 ± 5 | 2.2 ± 0.6 | 2.1 ± 0.4 | 138 ± 30 | 5.6 ± 0.3 | 2.1 ± 0.6 |
| **GTP** | 16 ± 2 | 2.0 ± 0.6 | 1.9 ± 0.4 | 44 ± 3 | 5.6 ± 0.3 | 1.3 ± 0.1 |
| **UTP** | 5.8 ± 1.0 | 2.3 ± 0.6 | 1.2 ± 0.1 | 12 ± 3 | 5.6 ± 0.2 | N/A |
| **CTP** | 2.2 ± 1.5 | 2.1 ± 0.6 | N/A | 1.3 ± 0.2 | 5.6 ± 0.1 | N/A |
| **ATP** | 0.8 ± 0.3 | 2.5 ± 0.7 | N/A | 1.4 ± 1 | 5.7 ± 0.3 | N/A |

* N/A = not applicable, as **Equation 1** was used for fitting which lacked the additional parameter *n*

# Supplementary Figures

# *
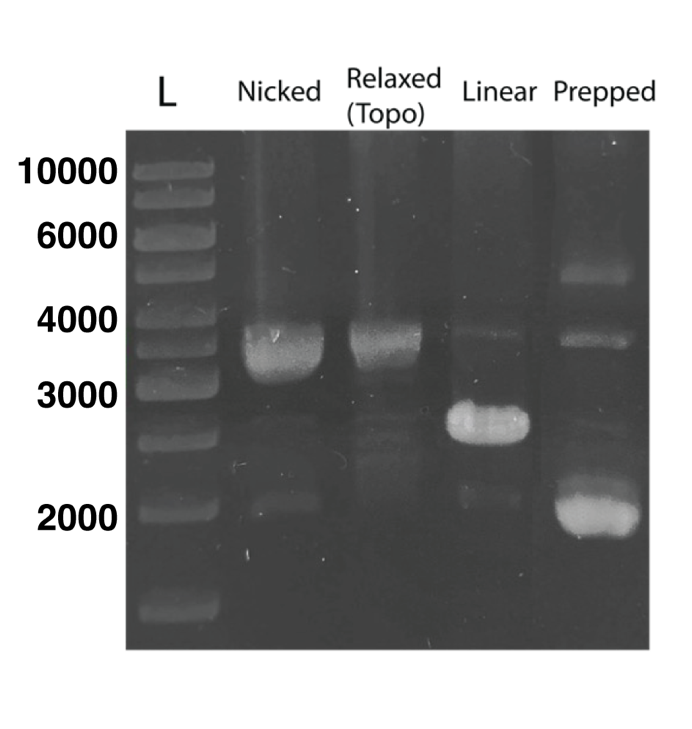
*

##### **Figure S1: Agarose gel of plasmid DNA templates.** A 0.8% agarose gel with lanes as follows: (*L*) a molecular weight ladder; (*Nicked*) prepped plasmid site-specifically nicked using Nt.BsmAI nicking endonuclease (New England BioLabs; catalogue # R0121S); (*Relaxed*) prepped plasmid treated with Topoisomerase I (New England BioLabs; catalogue # M0301); (*Linear*) prepped plasmid digested using the ScaI-HF restriction endonuclease (New England BioLabs; catalogue # R3122); (*Prepped*) untreated purified plasmid (Qiagen Midi Prep Kit; catalogue #: 27106) propagated in *E. coli* DH10B cells used for the *in vitro* transcription reactions presented in this work. Plasmid size is 2557 bp. Most of the prepped plasmid is found in the quickly migrating band compared to linear and relaxed samples, indicating superhelicity and torsional constraint.


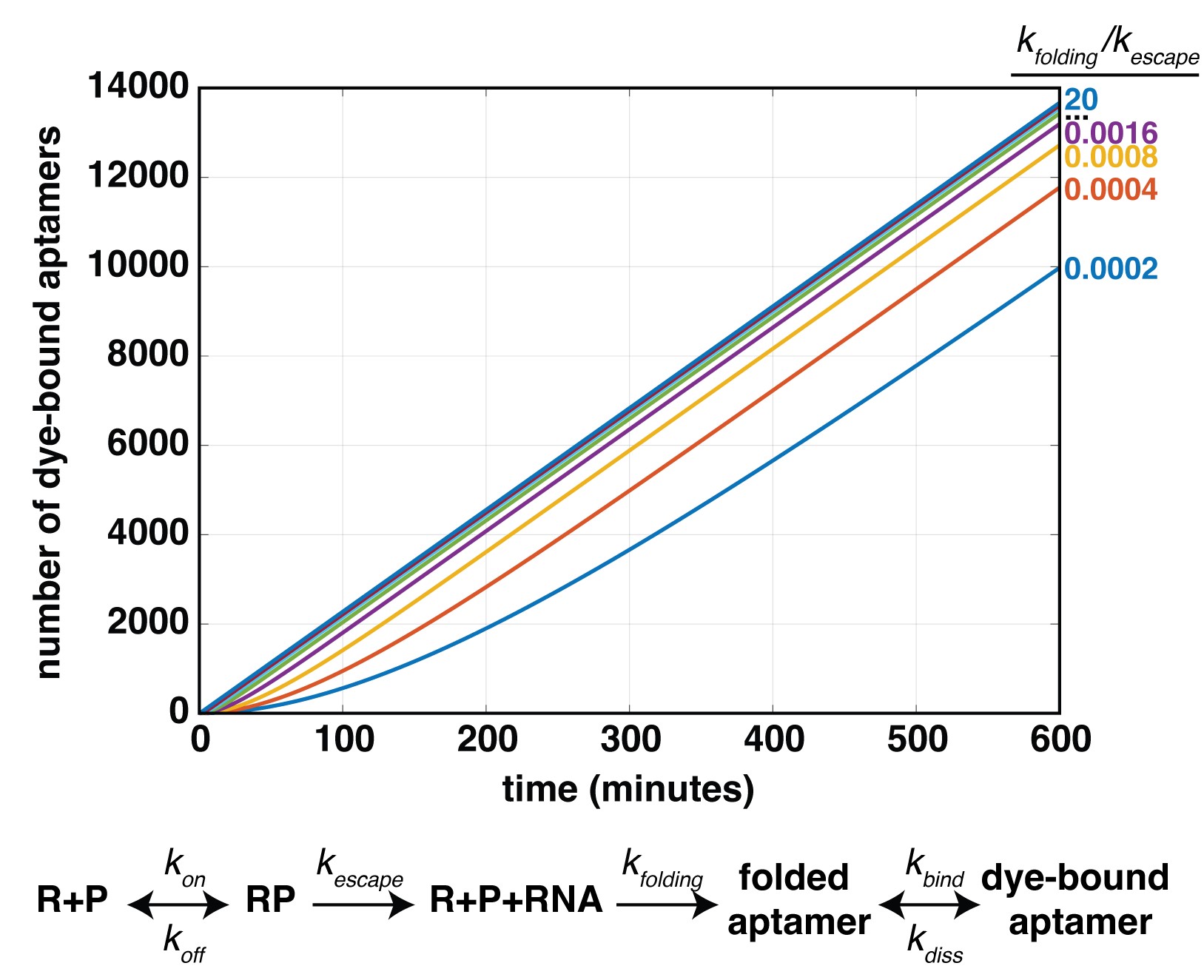


**Figure S2: Simulations where aptamer-folding is rate-limiting.** Simulations of transcription and fluorescence detection were performed according to the kinetic scheme above (**Supplementary Materials and Methods**), where R is RNAP and P is the promoter template. To assess how a slow molecular process downstream of the promoter, such as aptamer folding (*k_folding_*), may affect the steady-state rate of initiation, the rate of folding was titrated from 0.0002x to 20x the rate of promoter escape. The resulting curves illustrate that the steady-state rate of dye-bound aptamers (linear slope) is independent of the aptamer folding rate. However, when *k*_folding_ is slower than *k*_escape_, an increase in the time to reach steady-state (i.e., a lag-time) is observed. We also note that we don’t explicitly model elongation as multiple RNAPs can elongate in parallel on a single template without limiting the rate of initiation. In addition, previous studies illustrated the insertion of up to 500 bp in sequence between the promoter and the aptamer sequence had no effect on the observed lag-time (10). The parameters used in the simulation were as follows: *k*_on_ = 0.01 nM^-1^s^-1^, *k*off = 0.1 s^-1^, *k*escape = 0.5 s^-1^, *k*folding = (0.0001 – 10 s^-1^), *k*bind = 0.00004 nM^-1^s^-1^, *k*diss = 0.001 s^-1^, [RNAP] = 200 nM, [dye] = 20 μM.

**
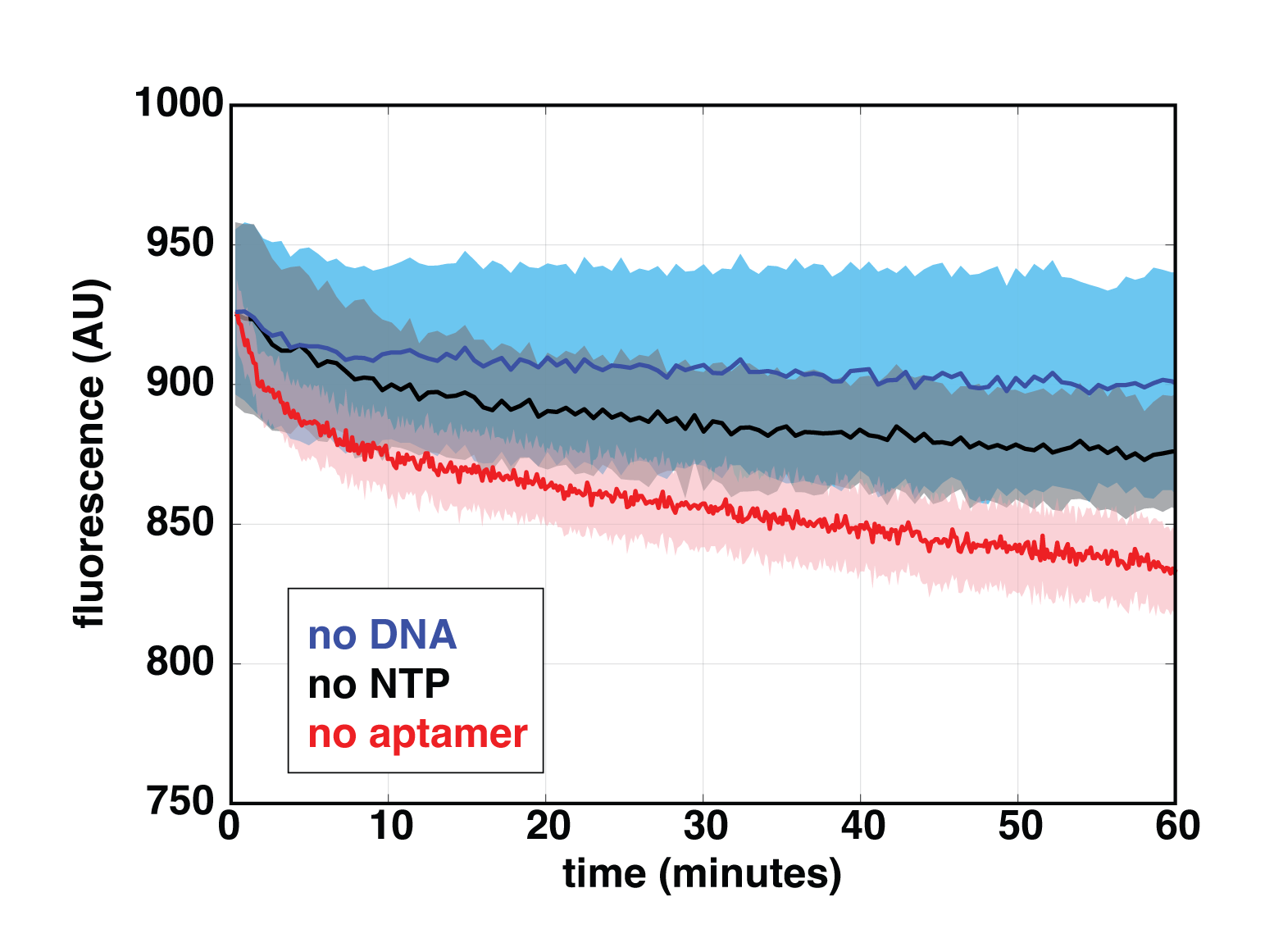
**

**Figure S3: Real-time fluorescent signal in the absence of aptamer formation.** Fluorescent signal was monitored as a function of time with 100 nM *Mtb* RNAP and 20 μM DFHBI under conditions where aptamer formation was prevented, either by leaving out 500 μM all NTPs (black), 5 nM *rrnA*P3 circular plasmid DNA (blue), or including the plasmid DNA template which lacked the aptamer sequence (red). Leaving out any one of these reagents results in no increase in fluorescence, but rather only a slow decay. As a result, in each data acquisition, a negative control is collected and subtracted from the experimental data to correct for this decay. These curves are similar, but there can be small variations of the shape and amplitude of the decay. As a result, we always set-up a negative control for each independent experiment which corresponded to the zero concentration of whatever reagent (NTPs or DNA) was used to initiate the reaction. While this correction doesn’t significantly affect moderate to high signal experiments (i.e., it only represents ~1% of the signal change observed at saturating NTPs), it becomes crucial when studying systems with low signals.


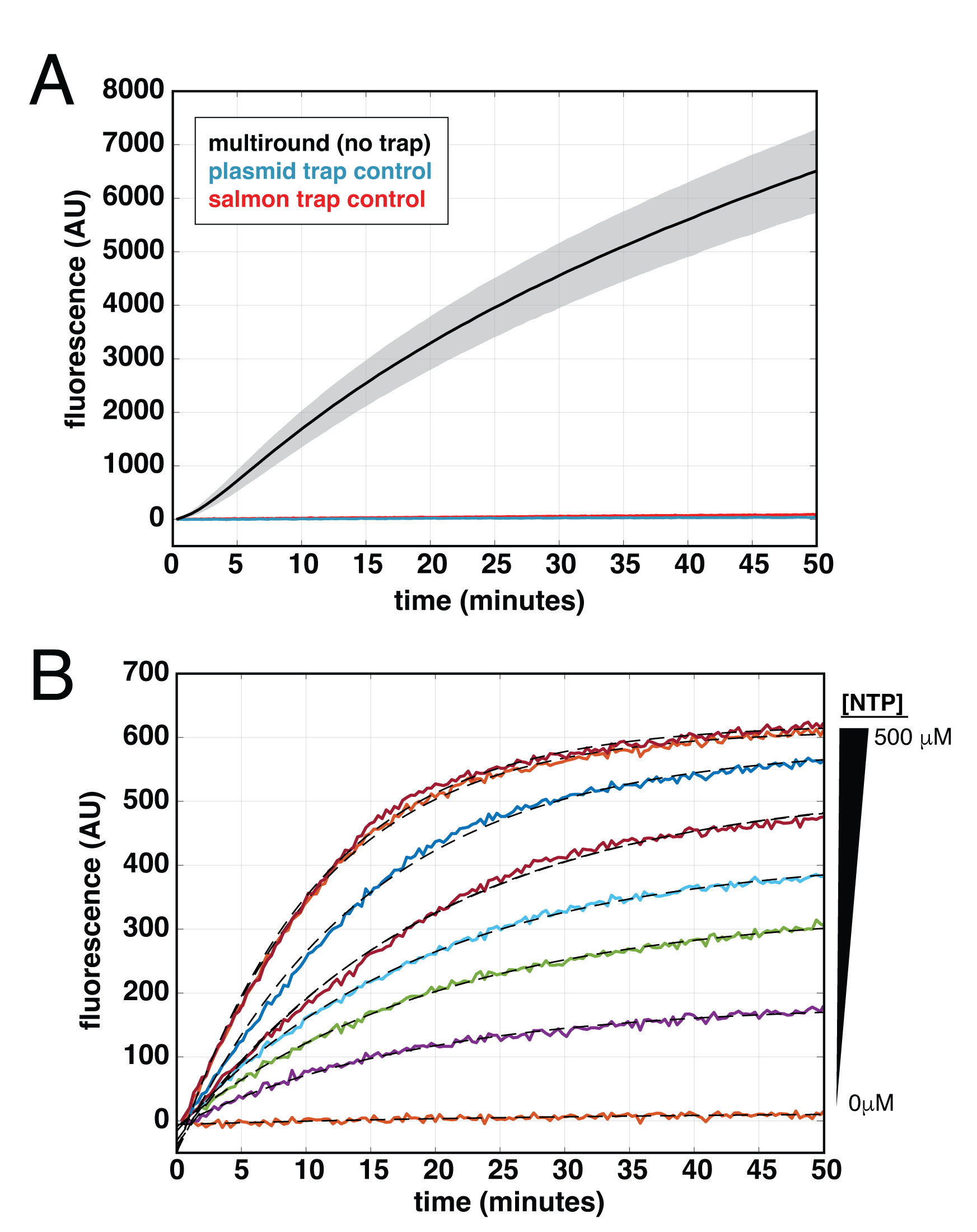


**Figure S4: Single-round trap controls and kinetics. (A)** Incubation of either 75 µg/mL of salmon-sperm DNA (red) or 150 nM “without aptamer” circular plasmid DNA (blue) trap prior to the addition of NTPs results in the complete abrogation of fluorescent signal, compared to conditions of 100 nM *Mtb* RNAP, 5 nM *Mtb* *rrnA*P3 circular plasmid DNA and 500 μM all NTPs without trap pre-incubation (black). **(B)** Titration of all NTPs under single-round conditions, where pre-incubated RNAP-promoter complexes (100 nM *Mtb* RNAP, 5 nM *Mtb* *rrnA*P3 circular plasmid DNA) were initiated with various concentrations of all NTPs and 75 µg/mL of salmon-sperm DNA. Dashed lines represent single-exponential fits at each NTP concentration tested. Here, fluorescence amplitude increases with increasing NTP concentration, as expected for a system that is prone to dissociation during promoter escape at low NTP concentrations.

**
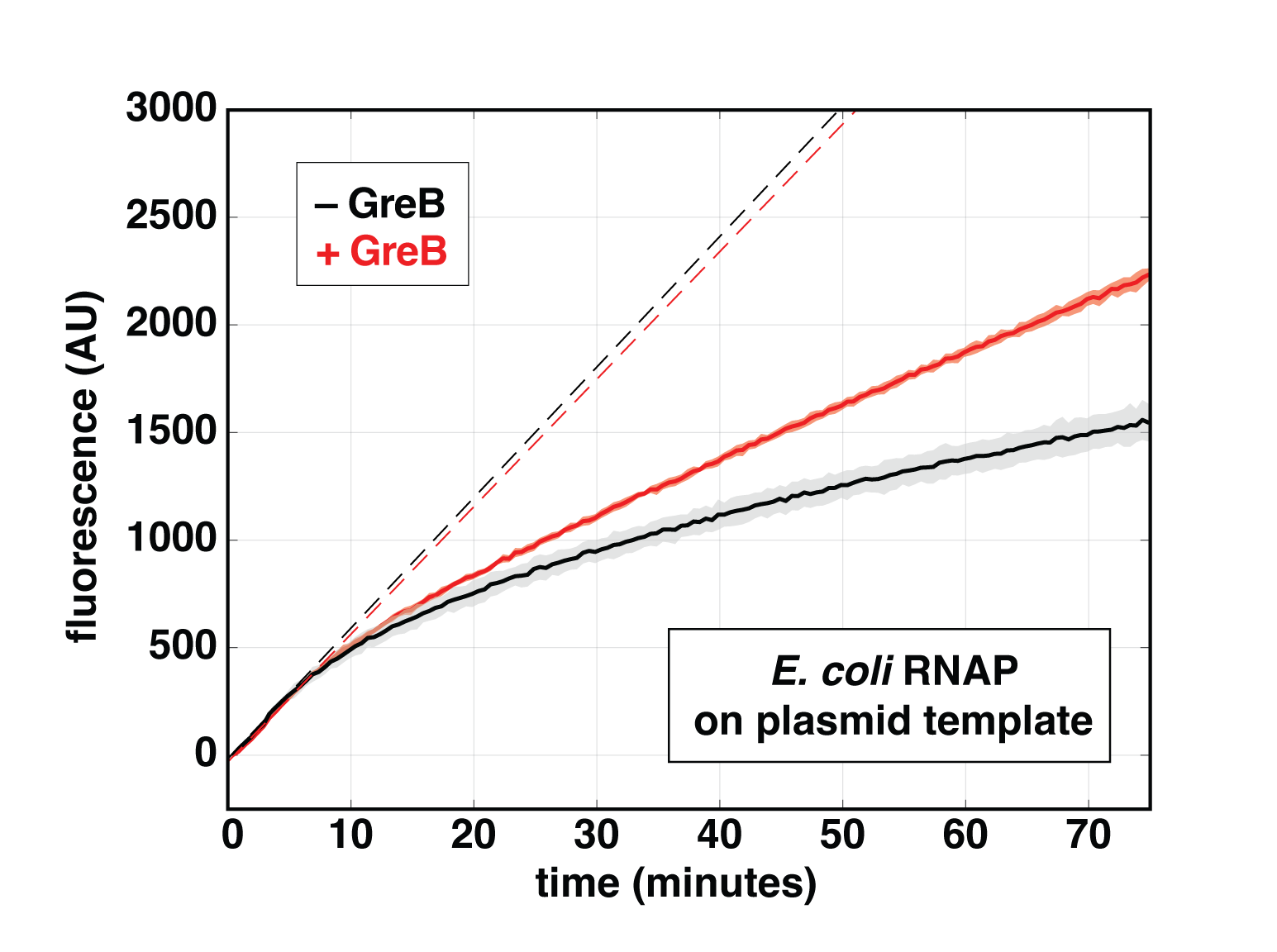
**

**Figure S5: GreB affects the long-term behaviour of fluorescent traces.** Experiments were performed using 100 nM *E. coli* σ^70^ RNAP, 500 μM all NTPs, 5 nM *Mtb* *rrnA*P3 circular plasmid DNA in the presence and absence of 1 μM *E. coli* GreB. Dotted lines represent the fits to the initial steady-state regime occurring from 0 – 10 minutes. The effect of GreB can be seen at longer time-scales past the initial steady-state.

**
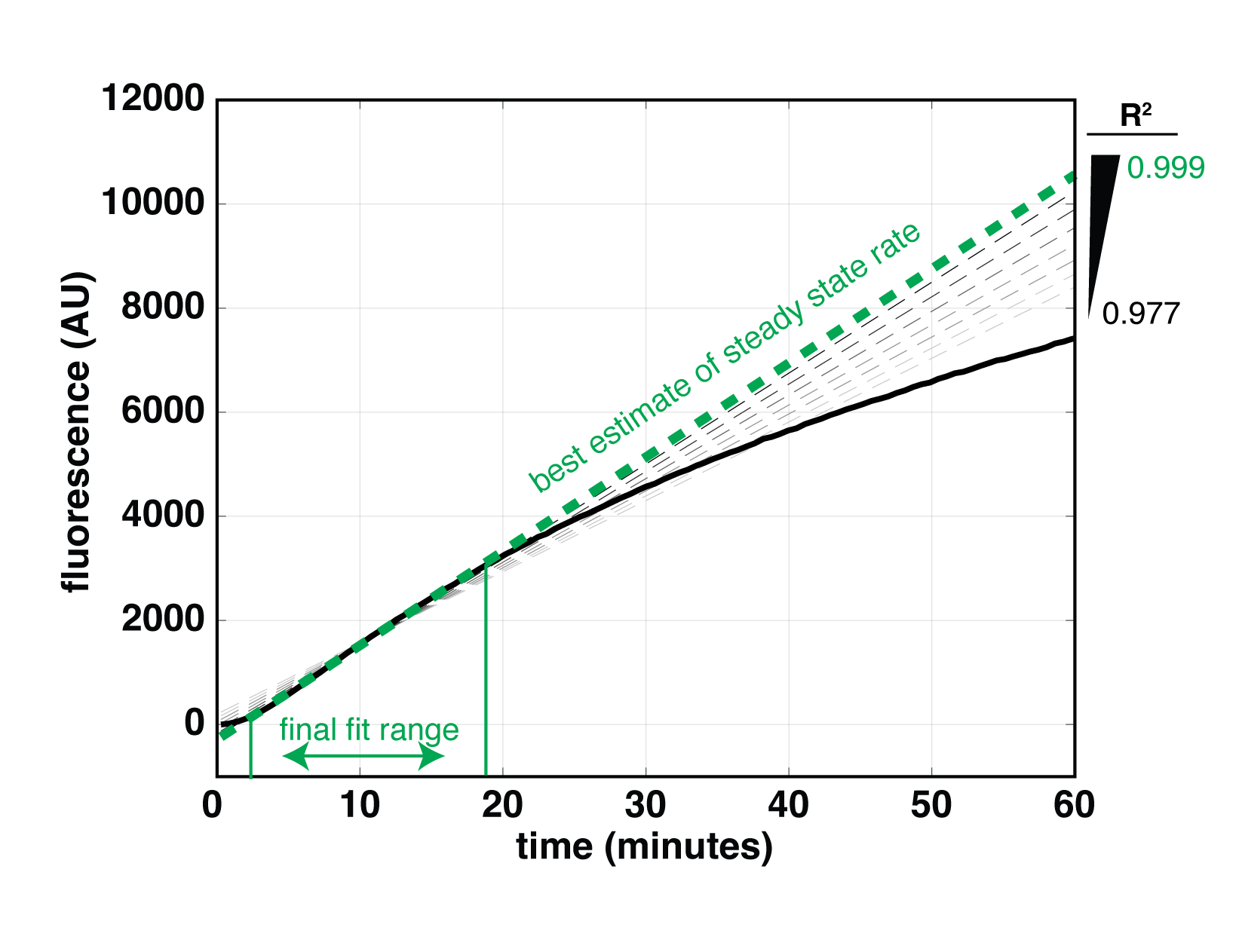
**

**iteration 1**

**data fit from 2.0333 to 59.7833; with 99 data points**

**fit rate = 136.7286**

**Rsquare = 0.97722**

**iteration 2**

**data fit from 2.0333 to 53.95; with 89 data points**

**fit rate = 142.0661**

**Rsquare = 0.9804**

**iteration 3**

**data fit from 2.0333 to 48.1167; with 79 data points**

**fit rate = 147.7899**

**Rsquare = 0.9837**

**iteration 4**

**data fit from 2.0333 to 42.2833; with 69 data points**

**fit rate = 153.9694**

**Rsquare = 0.98728**

**iteration 5**

**data fit from 2.0333 to 36.45; with 59 data points**

**fit rate = 160.7064**

**Rsquare = 0.99103**

**iteration 6**

**data fit from 2.0333 to 30.6167; with 49 data points**

**fit rate = 167.7945**

**Rsquare = 0.99465**

**iteration 7**

**data fit from 2.0333 to 24.7833; with 39 data points**

**fit rate = 174.7308**

**Rsquare = 0.99762**

**iteration 8**

**data fit from 2.0333 to 18.95; with 29 data points**

**fit rate = 180.8337**

**Rsquare = 0.9993**

**Figure S6: Overview of variable-time, iterative linear fitting approach.** Examples of recursive linear fitting using our code (https://github.com/egalburt/aptamer-flux-fitting) to illustrate how goodness of fit can change depending on the time interval. With each iteration, a shorter segment of the data is fit, and both the measured rate and R^2^ value increase until a threshold R^2^ is reached (in this case, 0.999).


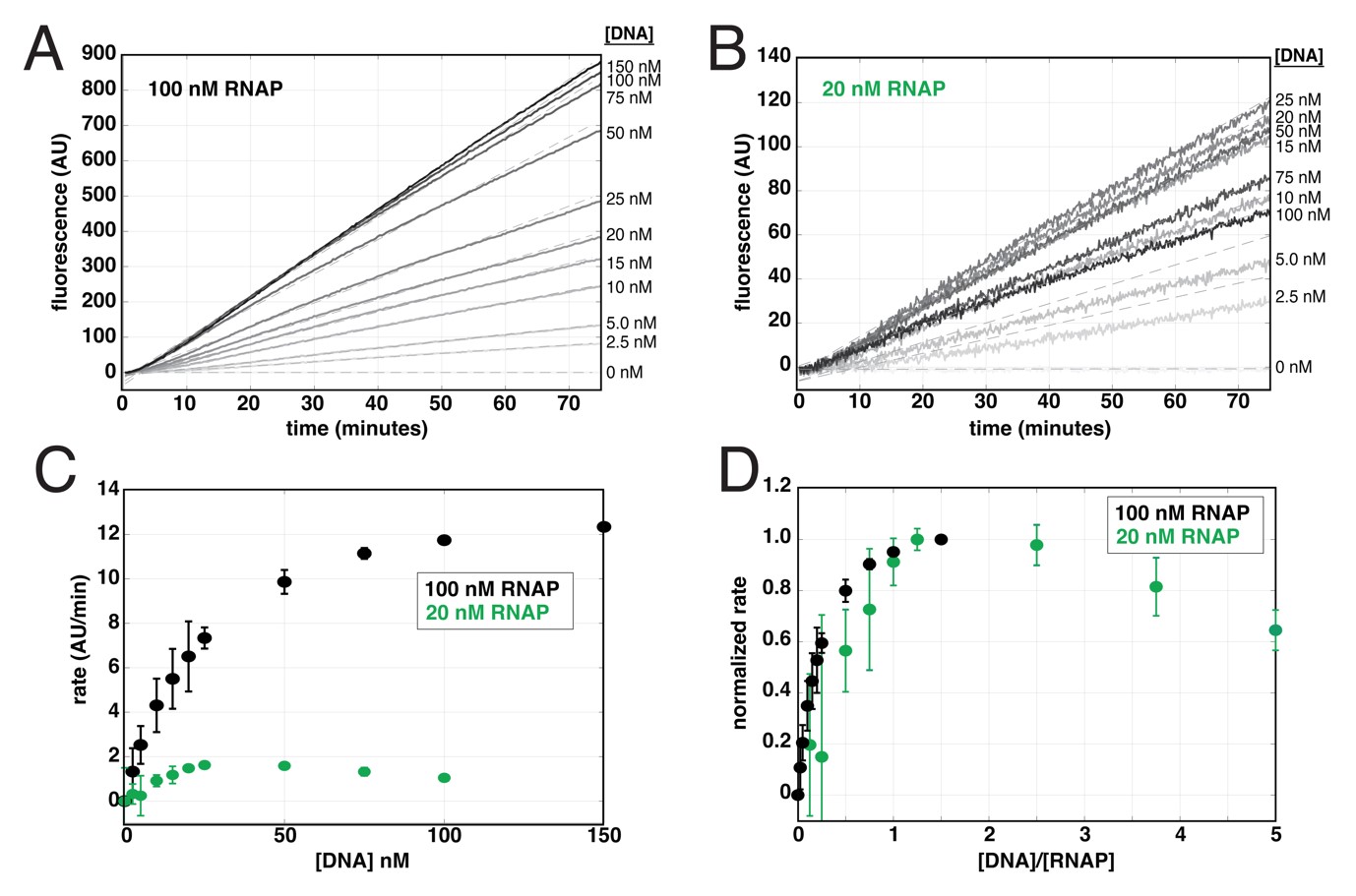


**Figure S7: Real-time data for DNA titrations on linear PCR templates.** Compare to circular plasmid data in **Figure 4** of the main text. Data was obtained at 500 μM all NTPs, titrating *Mtb* *rrnA*P3 linear PCR DNA template (2.5 – 150 nM) at either **(A)** 100 nM or **(B)** 20 nM *Mtb* RNAP concentrations**.** The unbiased linear fits of the early times are shown in grey dotted lines for each trace. **(C)** Steady-state rates obtained from the linear fits in (A) and (B) for 20 nM (green) and 100 nM (black) RNAP, plotted as a function of *rrnA*P3 linear PCR DNA concentration. **(D)** Steady-state rates, normalized from zero to one based on the lowest and highest rate obtained at each RNAP concentration, plotted as a function of the ratio of [DNA]:[RNAP] concentrations.


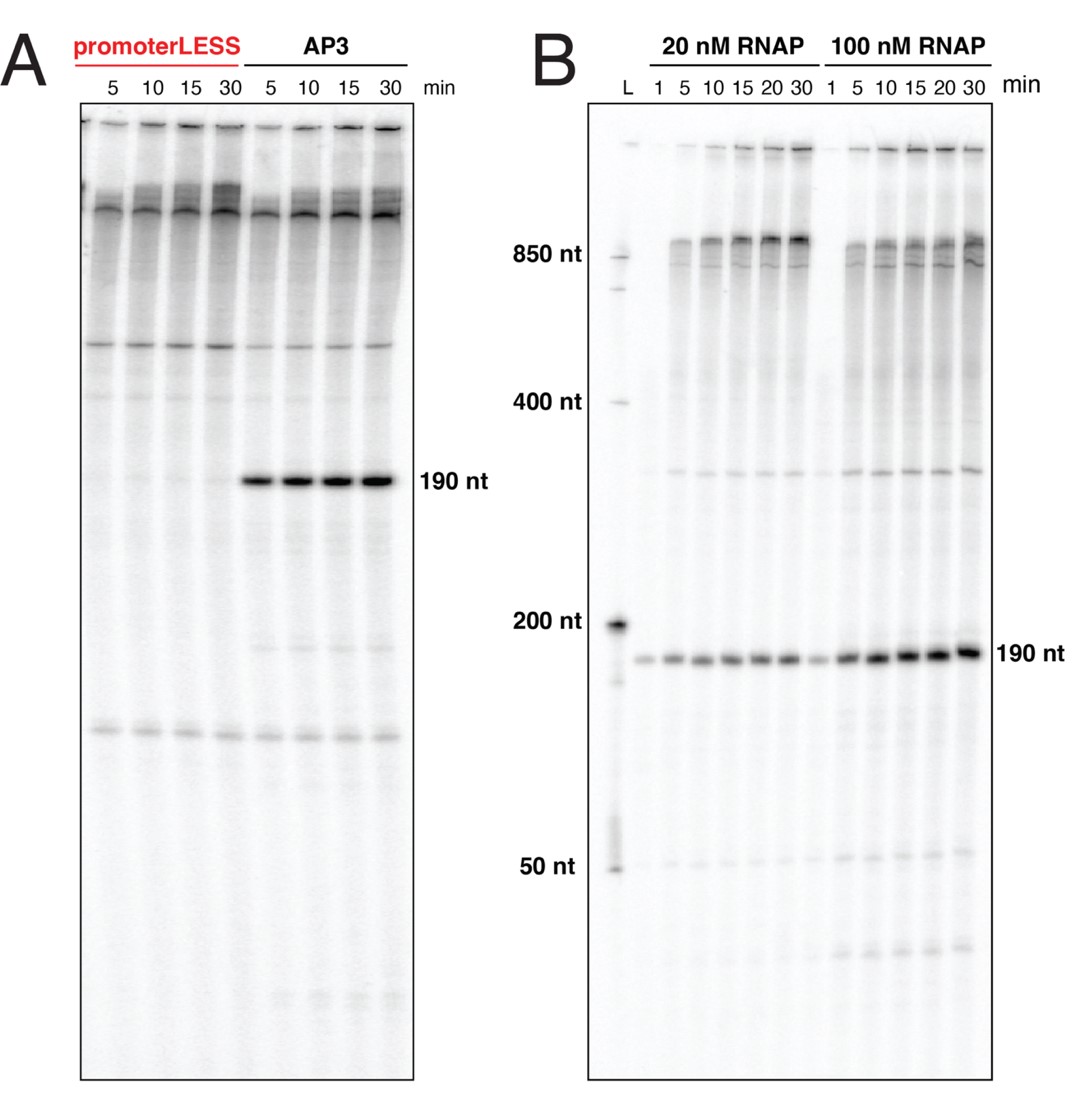


**Figure S8: Full gel images of the data presented in main text Figure 5. (A)** Time-dependent monitoring of all transcription bands observed with the *rrnA*P3 and promoterLESS circular plasmids and **(B)** with the *rrnA*P3 circular plasmid at 20 and 100 nM RNAP concentrations. All reactions were performed with *Mtb* RNAP, 5 nM circular plasmid DNA templates, 500 μM all NTPs and 20 μM DFHBI. The promoter-derived band containing the aptamer sequence runs to a length of ~ 190 nt.


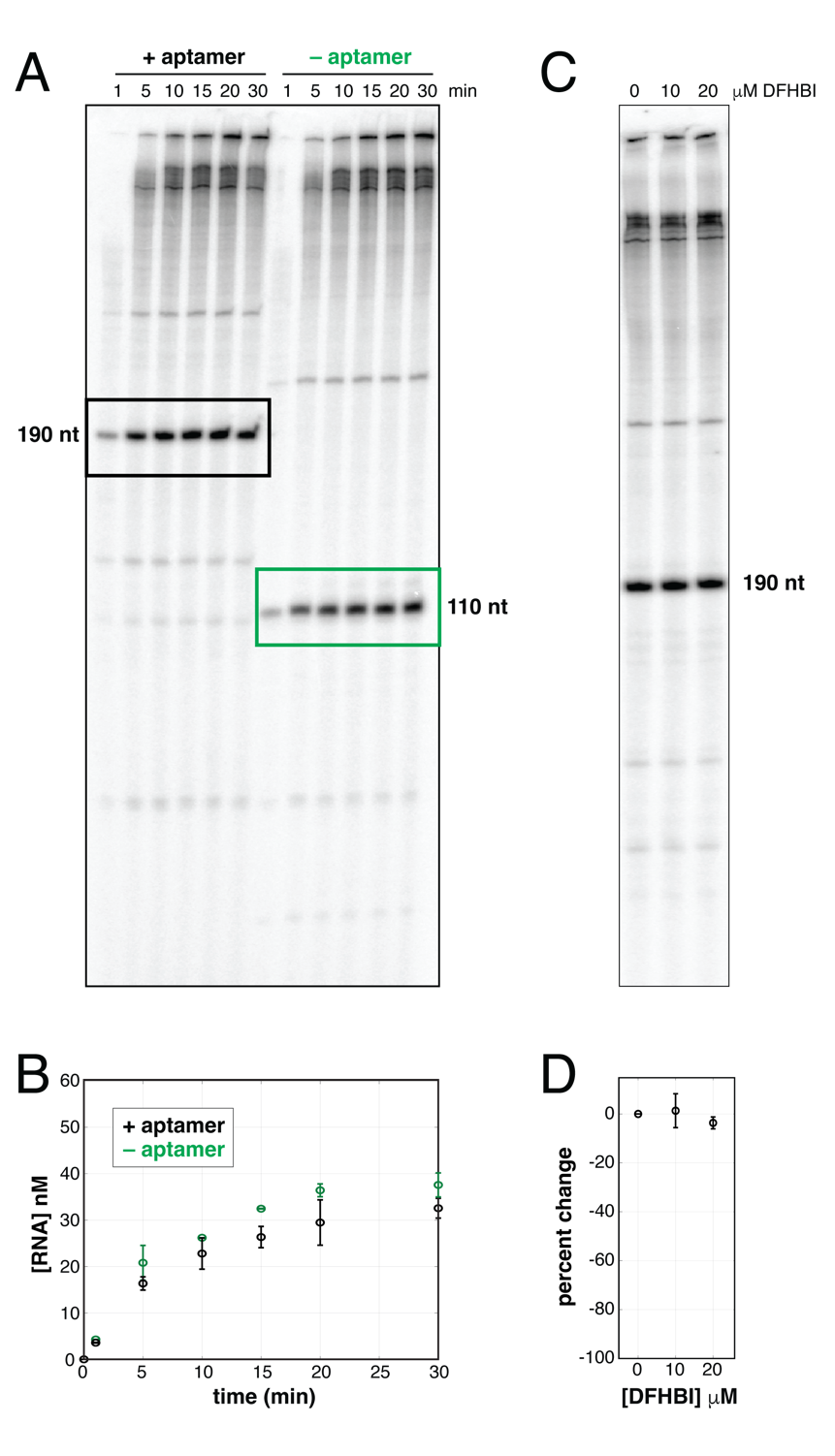


**Figure S9: Gel-based analysis of the effect of aptamer sequence and DFHBI dye. (A)** Time-dependent monitoring of all transcription bands observed with the *rrnA*P3 circular plasmids with and without the aptamer. **(B)** Band quantification in units of nM RNA of the promoter-derived product in (A) plotted as a function of time. **(C)** Measurement of all transcription bands observed with the *rrnA*P3 circular plasmid post 30 min after reaction initiation as a function of DFHBI concentration. **(D)** Band quantification of the promoter-derived product in (C) normalized to the signal obtained with 0 μM dye. Note that in (A) the promoter-derived band from the no-aptamer circular plasmid runs lower, as that 80 nt sequence was removed (**Supplementary Table 1**).


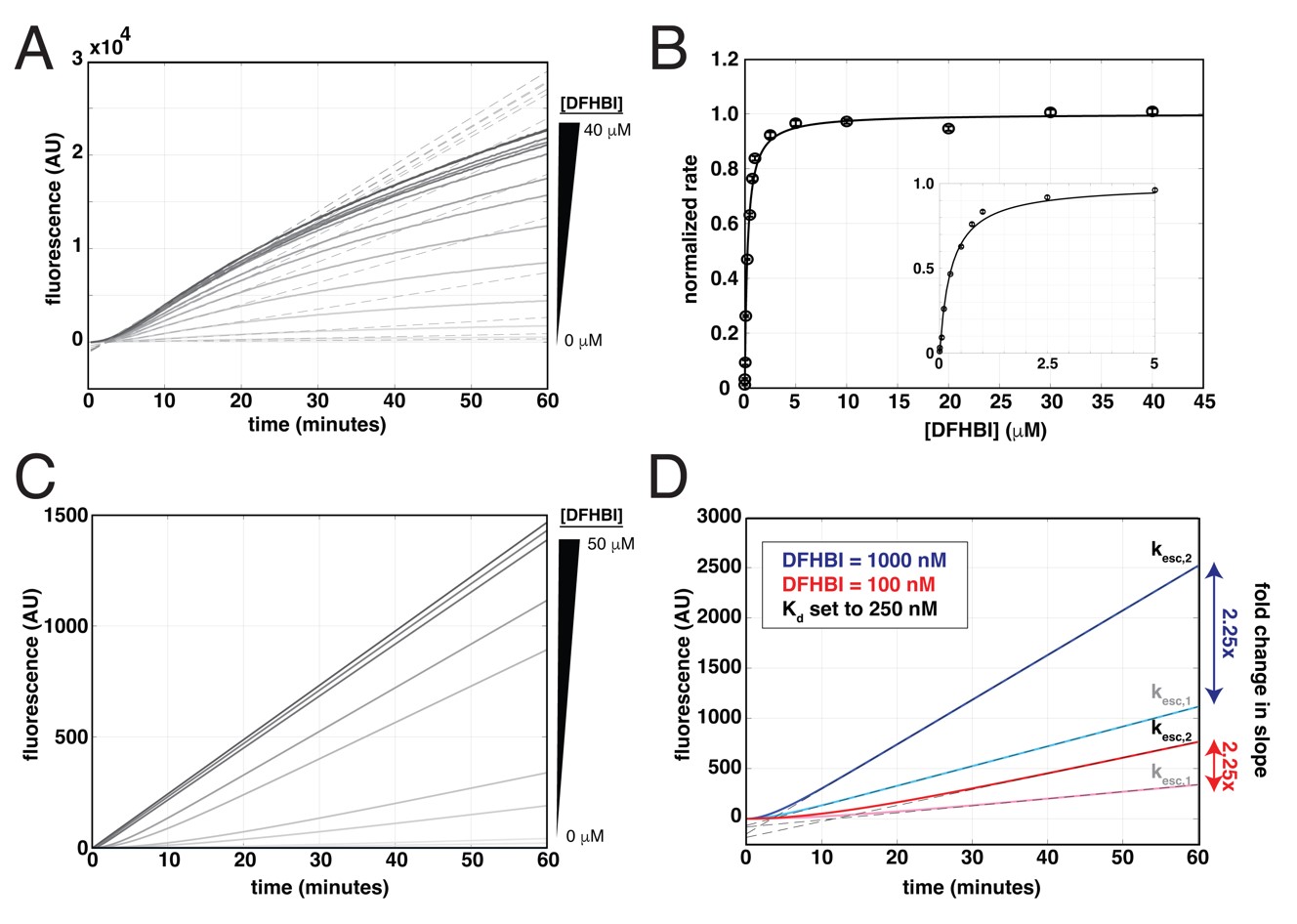


**Figure S10: Concentration dependencies of DFHBI on the fluorescent-aptamer signal. (A)** DFHBI dye titration with 100 nM *Mtb* RNAP, 500 µM NTPs, and 5 nM *rrnA*P3 circular plasmid template. The unbiased linear fits of the early times are shown in grey dotted lines for each trace. **(B)** Normalized steady-state rates plotted as a function of DFHBI concentration. Fit to a hyperbolic function yielded a midpoint of 270 nM, comparable to the binding affinities measured for Spinach, Spinach2, and Broccoli (12, 14, 24). Inset in (B) depicts the titration, from 0 – 5 μM dye concentrations. The steady-state rates measured in this regime change as the fraction of transcribed aptamer that is bound by dye changes. However, the underlying rate of transcription does not change as can be seen via the gel-based experiments in **Supplementary** **Figure S9C,D**. In essence then, dye concentration simply allows one to titrate the gain on the signal. **(C)** Kinetic simulations were performed using the model in **Supplementary Figure 2** with fixed rate constants and varying concentrations of DFHBI dye (**Supplementary Materials and Methods**). As seen in our experimental data, increasing dye concentration increases the slope of the curves until saturating at higher concentrations. **(D)** A comparison of the same kinetic scheme with two different escape rates (*k*_escape,1_ = 3X *k*_escape,2_) in the presence of two different dye concentrations (cyan/blue curves and pink/red curves). Although the absolute slopes of the curves at higher dye concentration are steeper, the fold change in the slopes between the two kinetic schemes remains the same. This illustrates the importance of making comparisons using the same dye concentrations, but also demonstrates the independence of mechanistic conclusions regarding initiation from the absolute concentration of dye.


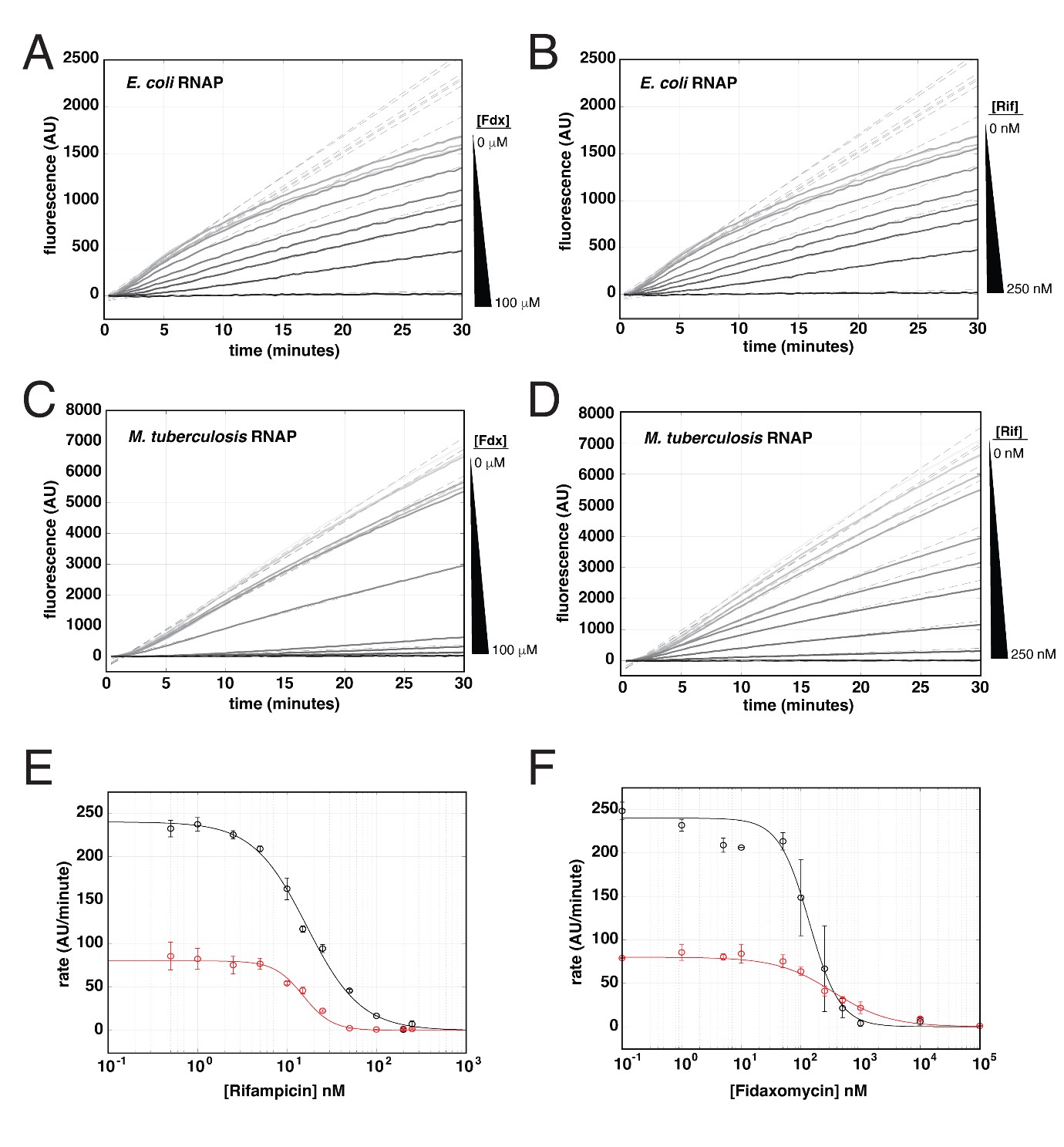


**Figure S11: Real-time data for antibiotic titrations and unnormalized *IC_50_* fits.** Real-time data for Fidaxomicin and Rifampicin titrations (**Supplementary Materials and Methods**) shown for *E. coli* (**A**, **B**) and *Mtb* (**C**, **D**) RNAPs. Dotted lines in (A-D) represent linear fits to the steady-state regime. The resulting steady-state rates are plotted as a function of Rifampicin (**E**) and Fidaxomicin (**F**) concentration for *Mtb* (black) and *E. coli* (red) RNAPs and are fit to **Equation 3** for a measure of the respective *IC_50_*s. Data in **Figure 8** is normalized from one to zero based on the fits presented in (E) and (F).

# Supplementary References

1. Lee,J., Vogt,C.E., McBrairty,M. and Al-Hashimi,H.M. (2013) Influence of Dimethylsulfoxide on RNA Structure and Ligand Binding. *Anal Chem*, 85, 9692–9698.

2. Stumper,S.K., Ravi,H., Friedman,L.J., Mooney,R.A., Corrêa,I.R., Gershenson,A., Landick,R. and Gelles,J. (2019) Delayed inhibition mechanism for secondary channel factor regulation of ribosomal RNA transcription. *Elife*, 8, e40576.

3. Furman,R., Tsodikov,O.V., Wolf,Y.I. and Artsimovitch,I. (2013) An Insertion in the Catalytic Trigger Loop Gates the Secondary Channel of RNA Polymerase. *J Mol Biol*, 425, 82–93.

4. Tetone,L.E., Friedman,L.J., Osborne,M.L., Ravi,H., Kyzer,S., Stumper,S.K., Mooney,R.A., Landick,R. and Gelles,J. (2017) Dynamics of GreB-RNA polymerase interaction allow a proofreading accessory protein to patrol for transcription complexes needing rescue. *Proc National Acad Sci*, 114, E1081–E1090.

5. Rammohan,J., Ruiz Manzano,A., Garner,A.L., Stallings,C.L. and Galburt,E.A. (2015) CarD stabilizes mycobacterial open complexes via a two-tiered kinetic mechanism. *Nucleic Acids Res*, 43, 3272–3285.

6. Rammohan,J., Ruiz Manzano,A., Garner,A.L., Prusa,J., Stallings,C.L. and Galburt,E.A. (2016) Cooperative stabilization of Mycobacterium tuberculosis rrnAP3 promoter open complexes by RbpA and CarD. *Nucleic Acids Res*, 44, 7304–7313.

7. Höfer,K., Langejürgen,L.V. and Jäschke,A. (2013) Universal Aptamer-Based Real-Time Monitoring of Enzymatic RNA Synthesis. *J Am Chem Soc*, 135, 13692–13694.

8. Nies,P. van, Canton,A.S., Nourian,Z. and Danelon,C. (2015) Chapter Ten Monitoring mRNA and Protein Levels in Bulk and in Model Vesicle-Based Artificial Cells. *Methods Enzymol*, 550, 187–214.

9. Nies,P. van, Nourian,Z., Kok,M., Wijk,R. van, Moeskops,J., Westerlaken,I., Poolman,J.M., Eelkema,R., Esch,J.H. van, Kuruma,Y., *et al.* (2013) Unbiased Tracking of the Progression of mRNA and Protein Synthesis in Bulk and in Liposome‐Confined Reactions. *Chembiochem*, 14, 1963–1966.

10. Huang,Y.-H., Trapp,V., Puro,O., Mäkinen,J.J., Metsä-Ketelä,M., Wahl,M.C. and Belogurov,G.A. (2022) Fluorogenic RNA aptamers to probe transcription initiation and co-transcriptional RNA folding by multi-subunit RNA polymerases. *Methods Enzymol*, 675, 207–233.

11. Okuda,M., Fourmy,D. and Yoshizawa,S. (2017) Use of Baby Spinach and Broccoli for imaging of structured cellular RNAs. *Nucleic Acids Res*, 45, 1404–1415.

12. Strack,R.L., Disney,M.D. and Jaffrey,S.R. (2013) A superfolding Spinach2 reveals the dynamic nature of trinucleotide repeat–containing RNA. *Nat Methods*, 10, 1219–1224.

13. Song,W., Strack,R.L., Svensen,N. and Jaffrey,S.R. (2014) Plug-and-Play Fluorophores Extend the Spectral Properties of Spinach. *J Am Chem Soc*, 136, 1198–1201.

14. Filonov,G.S., Moon,J.D., Svensen,N. and Jaffrey,S.R. (2014) Broccoli: Rapid Selection of an RNA Mimic of Green Fluorescent Protein by Fluorescence-Based Selection and Directed Evolution. *J Am Chem Soc*, 136, 16299–16308.

15. Autour,A., Westhof,E. and Ryckelynck,M. (2016) iSpinach: a fluorogenic RNA aptamer optimized for in vitro applications. *Nucleic Acids Res*, 44, 2491–2500.

16. Mitra,J. and Ha,T. (2019) Nanomechanics and co-transcriptional folding of Spinach and Mango. *Nat Commun*, 10, 4318.

17. Cawte,A.D., Unrau,P.J. and Rueda,D.S. (2020) Live cell imaging of single RNA molecules with fluorogenic Mango II arrays. *Nat Commun*, 11, 1283.

18. Roe,J.H. and Record,M.T. (1985) Regulation of the kinetics of the interaction of Escherichia coli RNA polymerase with the lambda PR promoter by salt concentration. *Biochemistry-us*, 24, 4721–6.

19. Leirmo,S., Harrison,C., Cayley,D.S., Burgess,R.R. and Record,M.T. (1987) Replacement of potassium chloride by potassium glutamate dramatically enhances protein-DNA interactions in vitro. *Biochemistry-us*, 26, 2095–2101.

20. Bera,S.C., America,P.P.B., Maatsola,S., Seifert,M., Ostrofet,E., Cnossen,J., Spermann,M., Papini,F.S., Depken,M., Malinen,A.M., *et al.* (2022) Quantitative parameters of bacterial RNA polymerase open-complex formation, stabilization and disruption on a consensus promoter. *Nucleic Acids Res*, 10.1093/nar/gkac560.

21. Warner,K.D., Chen,M.C., Song,W., Strack,R.L., Thorn,A., Jaffrey,S.R. and Ferré-D’Amaré,A.R. (2014) Structural basis for activity of highly efficient RNA mimics of green fluorescent protein. *Nat Struct Mol Biol*, 21, 658–663.

22. Qin,W., Li,L., Yang,F., Wang,S. and Yang,G.-Y. (2022) High-throughput iSpinach fluorescent aptamer-based real-time monitoring of in vitro transcription. *Bioresour Bioprocess*, 9, 112.

23. Orosz,A., Boros,I. and Venetianer,P. (1991) Analysis of the complex transcription termination region of the Escherichia coli rrn B gene. *Eur J Biochem*, 201, 653–659.

24. Paige,J.S., Wu,K.Y. and Jaffrey,S.R. (2011) RNA Mimics of Green Fluorescent Protein. *Science*, 333, 642–646.

25. Barnes,W.M. (1994) PCR amplification of up to 35-kb DNA with high fidelity and high yield from lambda bacteriophage templates. *Proc National Acad Sci*, 91, 2216–2220.
